# G9a and Sirtuin6 epigenetically modulate host cholesterol accumulation to facilitate mycobacterial survival

**DOI:** 10.1371/journal.ppat.1011731

**Published:** 2023-10-23

**Authors:** Praveen Prakhar, Bharat Bhatt, Gaurav Kumar Lohia, Awantika Shah, Tanushree Mukherjee, Ullas Kolthur-Seetharam, Nagalingam R. Sundaresan, Raju S. Rajmani, Kithiganahalli Narayanaswamy Balaji

**Affiliations:** 1 Department of Microbiology and Cell Biology, Indian Institute of Science, Bangalore, Karnataka, India; 2 Department of Biological Sciences, Tata Institute of Fundamental Research, Mumbai, Maharashtra, India; 3 Centre for Infectious Disease Research, Indian Institute of Science, Bangalore–, Karnataka, India; University of Massachusetts Medical School, UNITED STATES

## Abstract

Cholesterol derived from the host milieu forms a critical factor for mycobacterial pathogenesis. However, the molecular circuitry co-opted by *Mycobacterium tuberculosis* (Mtb) to accumulate cholesterol in host cells remains obscure. Here, we report that the coordinated action of WNT-responsive histone modifiers G9a (H3K9 methyltransferase) and SIRT6 (H3K9 deacetylase) orchestrate cholesterol build-up in *in vitro* and *in vivo* mouse models of Mtb infection. Mechanistically, G9a, along with SREBP2, drives the expression of cholesterol biosynthesis and uptake genes; while SIRT6 along with G9a represses the genes involved in cholesterol efflux. The accumulated cholesterol in Mtb infected macrophages promotes the expression of antioxidant genes leading to reduced oxidative stress, thereby supporting Mtb survival. In corroboration, loss-of-function of G9a *in vitro* and pharmacological inhibition *in vivo*; or utilization of BMDMs derived from *Sirt6*^−/−^ mice or *in vivo* infection in haplo-insufficient *Sirt6*^*−/+*^ mice; hampered host cholesterol accumulation and restricted Mtb burden. These findings shed light on the novel roles of G9a and SIRT6 during Mtb infection and highlight the previously unknown contribution of host cholesterol in potentiating anti-oxidative responses for aiding Mtb survival.

## Author summary

*Mycobacterium tuberculosis* (Mtb) rewires the host molecular machinery and several cellular and immunological pathways to survive amidst its challenging microenvironments. Of these, deregulated lipid accumulation in host cells has been shown to play a critical role in the pathogenesis of Mtb. Biochemical analysis has indicated that cholesterol and cholesteryl esters form a major part of the accumulated lipids, and several reports support the relevance of cholesterol in aiding Mtb survival. Therefore, in this study, we aimed to determine the regulatory mechanisms that lead to cholesterol accumulation in host cells during Mtb infection. We find WNT signaling-dependent epigenetic factors G9a and SIRT6 to favor cholesterol accretion during Mtb infection by differentially regulating cholesterol biosynthesis/uptake and efflux genes. We found a distinct role of the accumulated cholesterol in the expression of antioxidant genes, that consequently leads to Mtb growth. Interestingly, perturbation of WNT, G9a, SIRT6, cholesterol accumulation genes or antioxidant genes, was found to restrict Mtb burden *in vitro* and lack of G9a or SIRT6 was also found to limit Mtb growth and lung pathology in an *in vivo* mouse model of tuberculosis (TB); thereby suggesting the targeting of these epigenetic factors as potential adjuvants for TB therapy.

## Introduction

*Mycobacterium tuberculosis* (Mtb) rewires host cellular machinery to subvert protective immune responses and achieve a secure and nutrient-rich niche. Emerging evidence highlights the implication of epigenetic factors in Mtb-driven tuning of gene expression to effectuate such immune evasion [1–3]. Reports suggest that the histone methyltransferase (HMT) EZH2 epigenetically down-modulates MHC-II presentation [4]; SET8 HMT governs immune processes such as apoptosis, oxidative stress, and cytokine secretion [5]; while certain mycobacterial proteins themselves gain access to host chromatin and modulate a plenitude of immune genes [6]. One of the classical features of Tuberculosis is the accumulation of neutral lipids, cholesterol, and cholesteryl esters to generate foamy macrophage (FM) phenotype [7, 8]. Interestingly, certain studies report that lipid droplets do not serve as an important source of nutrients for Mtb and hence, do not affect Mtb growth [9] and that inhibition of fatty acid oxidation restricts intracellular growth of Mtb via ROS production [10]. However, various studies provide contrary evidence, supporting the notion that the formation of FMs positively correlates with mycobacterial virulence, and the loss of lipids from these cells compromises mycobacterial survival. This not only limits nutrients but also curbs the requisite cues for altering hosts’ ER stress, survival pathways, and autophagy levels. [11–19]. In this context, cholesterol serves essential functions for Mtb in the acquisition of dormancy and resistance to antibiotics in both, *in vitro* and *in vivo* systems [14][20]. To our interest, previous literature has reported that host cells such as macrophages form a major source of cholesterol for intracellular Mtb [21] and possibly for extracellular Mtb released into the caseated or cavitated TB granuloma lesions [14].

However, information regarding the mechanisms regulating cholesterol accumulation in hosts during Mtb infection requires extensive investigation. Existing literature suggests that genes responsible for cholesterol biosynthesis and homeostasis are epigenetically governed by miRNAs (miR-33a, miR-185), histone deacetylases (HDAC3, SIRT2, SIRT6) and HMTs (G9a) under distinct conditions [22] [23]. Crucial roles for SIRTUINS during Mtb infection have been highlighted [24]. Amongst these, SIRT3 has been reported to promote antimycobacterial responses [24]. Contrastingly, SIRT2 has been shown to reduce Mtb burden [25] and activation of the nuclear Sirtuin SIRT1, restricts Mtb growth by augmenting autophagy [26]. SIRT1 has also been shown to be involved in lipid metabolism, stress response, anti-inflammatory response, and cellular senescence in diverse contexts [27–31]. However, the contribution of the other nuclear Sirtuin, i.e., SIRT6, during infections, specifically mycobacterial infection, has not been addressed so far. SIRT6 has been shown to be upregulated in Mtb infection-related transcriptome dataset [32]. SIRT6 mainly deacetylates at H3K9- and H3K56- leading to gene repression. It is known to be associated with life span, genome stability and tumorigenesis [33–36]. Strikingly, SIRT6 has been identified as a potential regulator of SREBP1/2 functions, a major transcription factor for cholesterol metabolism [37]. Therefore, we were piqued to unravel the epigenetic contribution of SIRT6 in accumulating cholesterol during Mtb infection. Additionally, evidence from the literature suggest that apart from acetylation, methylation of H3K9 (mono- and di-), conferred by G9a, imparts crucial epigenetic signatures for shaping immunological fates during various pathophysiological conditions, such as T cell differentiation, immunological memory, viral latency, and endotoxin tolerance [38–43]. With this premise, we focused on elucidating the interplay of H3K9 methylation and acetylation by G9a and SIRT6, respectively, in defining cholesterol accumulation during Mtb infection.

We found that Mtb induces the expression of G9a and SIRT6, which contribute to epigenetically driven differential expression of cholesterol biosynthesis, uptake, and efflux genes, thereby allowing cholesterol accumulation during infection. WNT signaling, that has earlier been implicated in cell proliferation, migration, immunological processes and in shaping immune responses during Mtb infection [44, 45], was found to govern the levels of G9a and SIRT6 in this study. Further, the accumulated cholesterol was found to aid in mycobacterial survival by promoting anti-oxidative factors. Loss-of-function of G9a using a pharmacological inhibitor and that of SIRT6 using *Sirt6*^*−/+*^ mice in an *in vivo* mouse TB model was found to hamper host cholesterol accumulation and restrict Mtb burden. This was also corroborated by lung histology, which indicated a reduced severity of TB-like pathology in mice lacking G9a and SIRT6 functions. Together, we experimentally demonstrate for the first time that G9a and SIRT6 are upregulated during Mtb infection; and in conjunction mediate TB pathogenesis by epigenetically reprogramming cholesterol accumulation. Besides, this study underscores the relevance of specific G9a and SIRT6 inhibitors as plausible anti-TB adjuvants.

## Results

### Interception of G9a and SIRT6 leads to restricted mycobacterial burden

**Fig 1:**
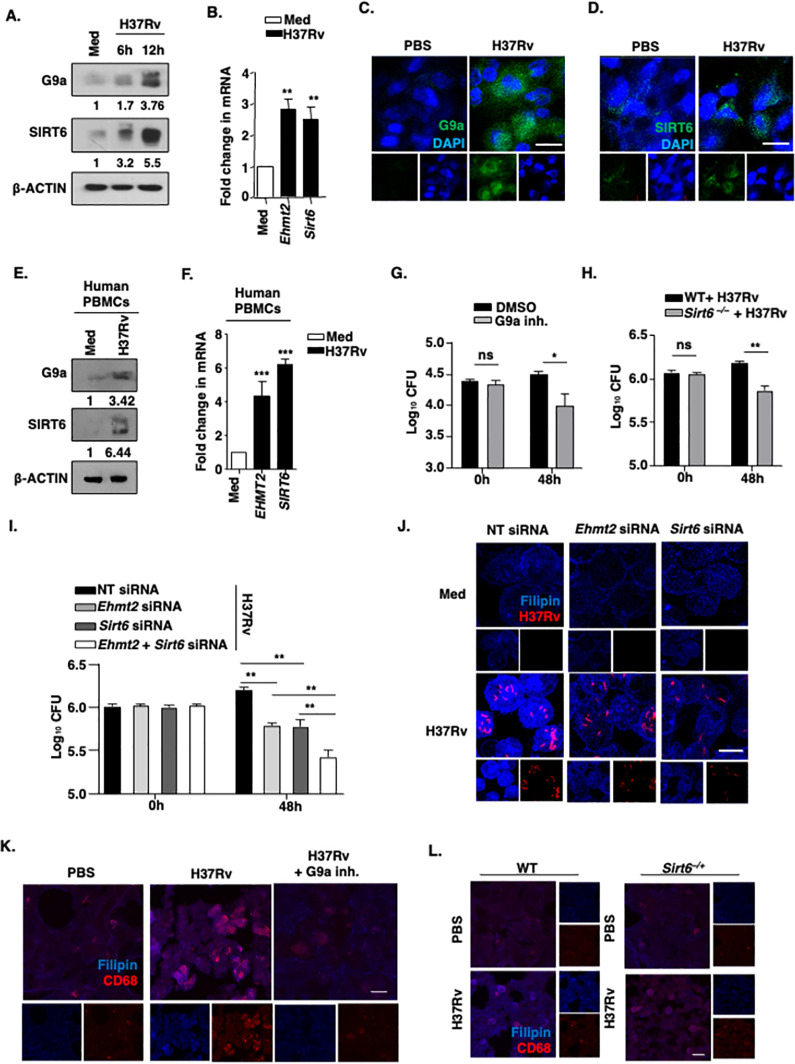
Interception of G9a and SIRT6 leads to restricted mycobacterial burden by modulating cholesterol accretion. **(A)** BALB/c peritoneal macrophages were infected with H37Rv for 6 and 12 h and **(A)** protein and **(B)** transcript level of G9a, SIRT6 were analyzed. **(C, D)** *In vivo* expression of G9a **(C)** and SIRT6 **(D)** was analyzed in lung cryosections of uninfected mice and mice infected with H37Rv for 56 days by immunofluorescence. **(E-F)** Human PBMCs were infected with H37Rv for 12 h and analyzed for G9a and SIRT6 at protein **(E)** and transcript **(F)** level. **(G-H)** *In vitro* CFU was assessed 48 h post H37Rv infection (MOI 1:5) under the following conditions: **(G)** in BALB/c mouse peritoneal macrophages treated with G9a inhibitor (5 μM) or **(H)** in BMDMs derived from WT (littermate control) or *Sirt6*^−/−^ mice or **(I)** in BALB/c mouse peritoneal macrophages transiently transfected with siRNAs against *Ehmt2* or *Sirt6* or both. **(J)** BALB/c mouse peritoneal macrophages transfected with NT or *Ehmt2* or *Sirt6* siRNA were assessed for free cholesterol level upon 48 h infection with tdTomato-expressing H37Rv by immunofluorescence. Representative images of Filipin stained macrophages. **(K)** Lung cryosections from uninfected or 56 days H37Rv-infected/ G9a inhibitor (40 mg/kg) treated BALB/c mice were assessed for free cholesterol by Filipin staining in macrophages stained with CD68. **(L)** Lung cryosections of uninfected and infected WT (littermate control) and *Sirt6*^*−/+*^ mice were assessed for free cholesterol levels by Filipin staining in macrophages stained by CD68. The MOI of infection is 1:10 (macrophage: mycobacteria) for all the *in vitro* experiments. All data represents mean ± SEM from 3 independent experiments; *, P < 0.05, **, P < 0.01; ns, not significant (Student’s t-test for B, F-H and One-way ANOVA for I) and the blots are representative of 3 independent experiments. Med, medium (uninfected/untreated cells maintained in DMEM supplemented with 10% heat inactivated FBS for the entire duration of the experiment); WT, wild type; inh., inhibitor; NT, non-targeting; BMDM, bone marrow derived macrophages; PBMC, peripheral blood mononuclear cells. Scale bar, 10μm.

We embarked on this study to understand how epigenetic factors, G9a and SIRT6 shape the course of Mtb infection. First, we found that Mtb H37Rv infection of mouse peritoneal macrophages induced the expression of the histone modifiers HMT G9a (encoded by *Ehmt2*) and HDAC SIRT6 (encoded by *Sirt6*) 12 h post infection at protein as well as transcript level **(Fig 1A and 1B).** Interestingly, enhanced expression of G9a and SIRT6 did not affect the global histone H3K9 monomethylation and H3K9 acetylation pattern respectively **(S1A Fig**). Corollary to our *in vitro* results, we observed augmented levels of G9a and SIRT6 at the transcript and protein level in mouse model of pulmonary Mtb infection (**S1B Fig and Fig 1C and 1D**). To show the translational importance of our study we further corroborated our findings in human primary macrophages. G9a and SIRT6 were significantly induced in human primary macrophages upon Mtb H37Rv infection at protein as well as transcript level (**Fig 1E and 1F**). Further, the induction of G9a and SIRT6 was found to be specific to virulent species of mycobacterium as infection of mouse peritoneal macrophages with the non-pathogenic *Mycobacterium smegmatis* showed only a weak expression of G9a and Sirt6 (**S1C Fig**). Collectively, we show that histone modifiers G9a and SIRT6 are significantly induced upon pathogenic Mtb H37Rv infection in mice and human macrophages.

To evaluate the possible contribution of these epigenetic factors to Mtb infection, *in vitro* CFU assays were performed. Inhibition of G9a using a specific pharmacological inhibitor (BIX-01294) was found to compromise Mtb H37Rv burden after 48 h of *in vitro* infection (**Fig 1G**). Also, mycobacterial CFU was assessed in BMDMs derived from WT (littermate control) and *Sirt6* knockout (Sirt6^−/−^) mice as the premature aging and death of *Sirt6*^−/−^ mice by 4 weeks postnatally [46] hinders the isolation of thioglycollate-elicited peritoneal macrophages and long-term *in vivo* experiments. SIRT6 expression was validated in the lung homogenate of WT, *Sirt6*^*−/+*^ and *Sirt6*^−/−^ mice **(S1D Fig)**. We found the Mtb H37Rv burden to be restricted in *Sirt6*^−/−^ BMDMs compared to the infected WT BMDMs (**Fig 1H**). Further, to elucidate the potential synergistic effect of G9a and SIRT6 on Mtb survival we performed *in vitro* CFU in peritoneal macrophages, by silencing *Ehmt2* or *Sirt6* individually or together. Individual silencing of G9a and SIRT6 lead to a compromised mycobacterial CFU, that was further diminished in mouse peritoneal macrophages knocked down for both *Ehmt2* and *Sirt6* (**Fig 1I)**. **S1E Fig**: knockdown validation; S1F Fig: cell viability was assessed upon G9a and SIRT6 knockdown using specific siRNAs and we did not observe any significant difference in cell viability. Interestingly, we also found that the expression of G9a and SIRT6 does not the depend on each other during Mtb infection (Data not shown). Collectively, these results suggest a critical role for the epigenetic modifiers G9a and SIRT6 in the successful survival of mycobacterium.

### G9a and SIRT6 effectuate cholesterol accumulation during mycobacterial pathogenesis

As described earlier, among various factors, host-derived cholesterol forms an integral part of mycobacterial pathogenesis *in vitro* and *in vivo*. In this context, virulent Mtb H37Rv infection was found to trigger cholesterol accumulation in host macrophages, unlike *M*. *smegmatis* infection, as assessed by Filipin staining **(S2A and S2B Fig)**. The same was mirrored in the lungs of mice infected with Mtb H37Rv, wherein staining lung cryosections with Filipin showed a significant increase in cholesterol accumulation specifically in macrophages **(S2C and S2D Fig)**. With the premise that both G9a and SIRT6 have been reported to epigenetically regulate cholesterol levels [22], we sought to explore their contribution to cholesterol accumulation in the context of Mtb infection. It was observed that *in vitro* silencing of *Ehmt2* and *Sirt6* via specific siRNAs led to a marked decline in the ability of Mtb H37Rv to furnish cholesterol accretion as assessed by Filipin staining **(Figs 1J and S2E)**. To assess for any alteration in cell viability due to siRNA treatment in the infection scenario, we performed MTT assay at 48hpi with and without G9a or SIRT6 knockdown using specific siRNAs and did not observe any significant difference in cell viability (**S2F Fig)**. We further strengthened our findings by using a cholesterol quantification kit that corroborated our Filipin staining results on G9a- and SIRT6-dependent cholesterol accumulation during Mtb infection. To negate the possibility that the observed reduction in cholesterol could be due altered cell viability, cholesterol estimation performed based on cell number (4 million each) **(S2G Fig, right panel)** was normalized to total cellular protein levels **(S2G Fig, left panel)** and the results consistently demonstrate an increase in cellular cholesterol levels upon H37Rv infection, which are effectively reduced upon the silencing of G9a and SIRT6. Also, significantly less cholesterol was detected by Filipin staining in BMDMs derived from *Sirt6*^*−/−*^ mice, even in the presence of Mtb H37Rv infection **(S2H and S2I Fig)**. Interestingly, *Sirt6*^*−/+*^ infected macrophages treated with G9a inhibitor (BIX-01294) showed further decrease in cholesterol accumulation upon H37Rv infection, indicating a collegial role of G9a and SIRT6 in driving Mtb H37Rv infection-induced cholesterol accumulation **(S2K and S2L Fig)**. Substantiating the same, macrophage-specific accumulation of cholesterol was reduced in the lungs of G9a inhibitor-treated mice **(Figs 1K and S2J)** and *Sirt6*^*−/+*^ mice **(Figs 1L and S2M)**. This evidence indicates the ability of Mtb to utilize G9a and SIRT6 for mediating the process of cholesterol accumulation in host cells.

### Cholesterol biosynthesis and transport genes are differentially regulated by G9a and SIRT6

The accumulation of cholesterol in a cell or tissue results from the coordinated interplay of genes involved in its biosynthesis **(S3A Fig)**, uptake and efflux. To this end, the status of the pertinent markers (23 genes) of each function was assessed for their transcript level expression during infection with Mtb H37Rv *in vitro* and *in vivo* **(S3B and S3C Fig)**. We observed that genes involved in cholesterol uptake (*Lrp2*) (denoted in grey across the figures) and biosynthesis (*Aacs*, *Hmgcs1*, *Mvd*, *Dhcr24*) were significantly upregulated during infection; while those implicated in efflux (*Abca1*, *Abcg1*) showed a marked downregulation. We further assessed the transcript levels of the altered genes in human PBMCs infected with Mtb H37Rv. Corroborating our *in vitro* and *in vivo* mice data, we observed a significant increase in the transcript levels of genes involved in cholesterol uptake (*LRP2*) and biosynthesis (*AACS*, *HMGCS1*, *MVD*, *DHCR24*) with a downregulation in the expression of efflux genes (*ABCA1*, *ABCG1*) in H37Rv-infected human PBMCs **(S3D Fig)**. These data show that Mtb alters the expression of cholesterol metabolism and transport genes.

**Fig 2:**
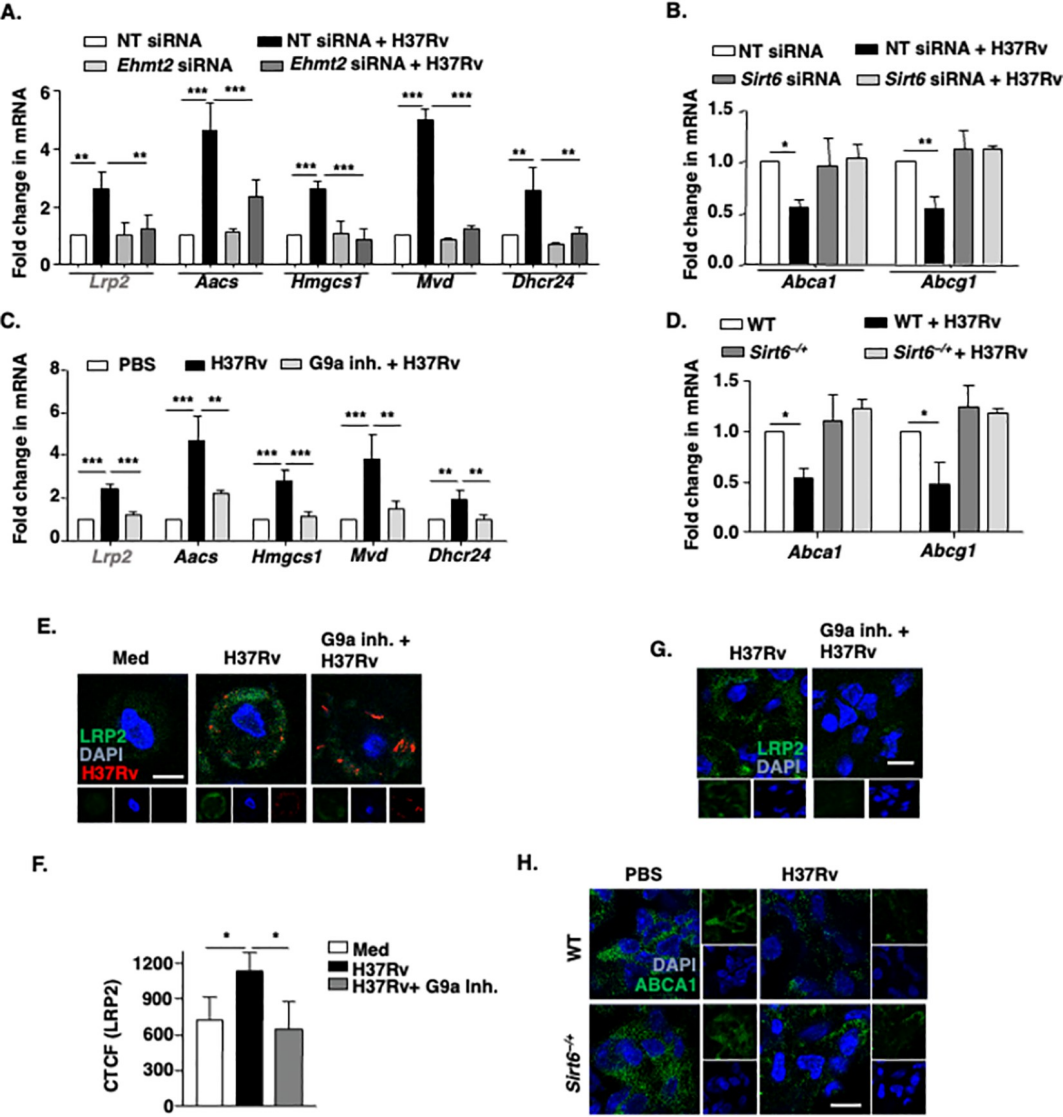
Cholesterol biosynthesis and transport genes are selectively regulated by G9a and SIRT6. **(A, B)** BALB/c mouse peritoneal macrophages were transfected with NT or *Ehmt2* or *Sirt6* siRNA. Transfected cells were infected with H37Rv for 12 h and the expression of the indicated genes were assessed by qRT-PCR. **(C, D)** RNA was isolated from the lung homogenates from the indicated groups of mice after 56 days of total infection and the transcript levels of the indicated cholesterol metabolism genes were analyzed by qRT-PCR. **(E, F)** Surface expression of LRP2 was analyzed by immunofluorescence in BALB/c peritoneal macrophages pre-treated with G9a specific inhibitor (5 μM) for 1 h followed by infection with tdTomato-expressing H37Rv for 12 h. **(E)** Representative images and **(F)** its quantification. **(G)** Lung cryosections from 56 days H37Rv-infected/G9a inhibitor (40 mg/kg) treated BALB/c mice were assessed for surface expression of LRP2 by immunofluorescence. **(H)** Lung cryosections of uninfected and 56 days H37Rv-infected WT (littermate control) and *Sirt6*^*−/+*^ mice were assessed for the protein level of ABCA1 by immunofluorescence. *In vivo* data represents the mean ± SEM from 2–3 mice. The MOI of infection is 1:10 (macrophage: mycobacteria) for all the *in vitro* experiments unless otherwise stated. All data represents the mean ± SEM from 3 independent experiments, *, P < 0.05; **, P < 0.01; ***, P < 0.001 (One-way ANOVA for A-D and F). Med, medium; WT, wild type; inh., inhibitor; NT, non-targeting. Scale bar, 10μm.

Next, we aimed to determine if the epigenetic modifiers G9a and SIRT6 have a role in regulating the expression of cholesterol metabolism genes. Interestingly, this differential gene expression was found to be finely tuned by the combined activities of G9a and SIRT6. We found that siRNA mediated depletion of G9a compromised the expression of the biosynthesis and uptake genes (*Lrp2*, *Aacs*, *Hmgcs*, *Mvd*, *Dhcr24*) at the transcript level **(Fig 2A)**. Further, siRNA-mediated knock-down of SIRT6 rescued the Mtb-dependent downregulation of cholesterol efflux genes (*Abca1*, *Abcg1*) **(Fig 2B)**. In line, overexpressing SIRT6 led to a marked decrease in *Abca1* and *Abcg1* expression **(S3E Fig)**. The transcript level profiling performed for the concerned genes in the lungs of mice treated with G9a inhibitor *in vivo* or *Sirt6*^*−/+*^ mice also yielded a similar pattern **(Fig 2C and 2D)**. These findings were validated at the protein level, where G9a inhibitor limited the surface expression of LRP2 during infection, both *in vitro* **(Fig 2E and 2F)** and *in vivo* **(Fig 2G)**; and ABCA1 protein expression was found to be elevated in the lungs of Mtb H37Rv-infected *Sirt6*^*−/+*^ mice **(Fig 2H)**, compared to that in the infected wild type controls. The protein level of ABCA1 was also rescued in macrophages knocked down for *Sirt6* **(S3F Fig)**. These sets of experiments show that Mtb H37Rv induced G9a and SIRT6 regulate the expression of cholesterol biosynthesis/ uptake and efflux genes, respectively.

### G9a-SREBP2 and SIRT6 transcriptionally fine-tune cholesterol accumulation during Mtb infection

**Fig 3:**
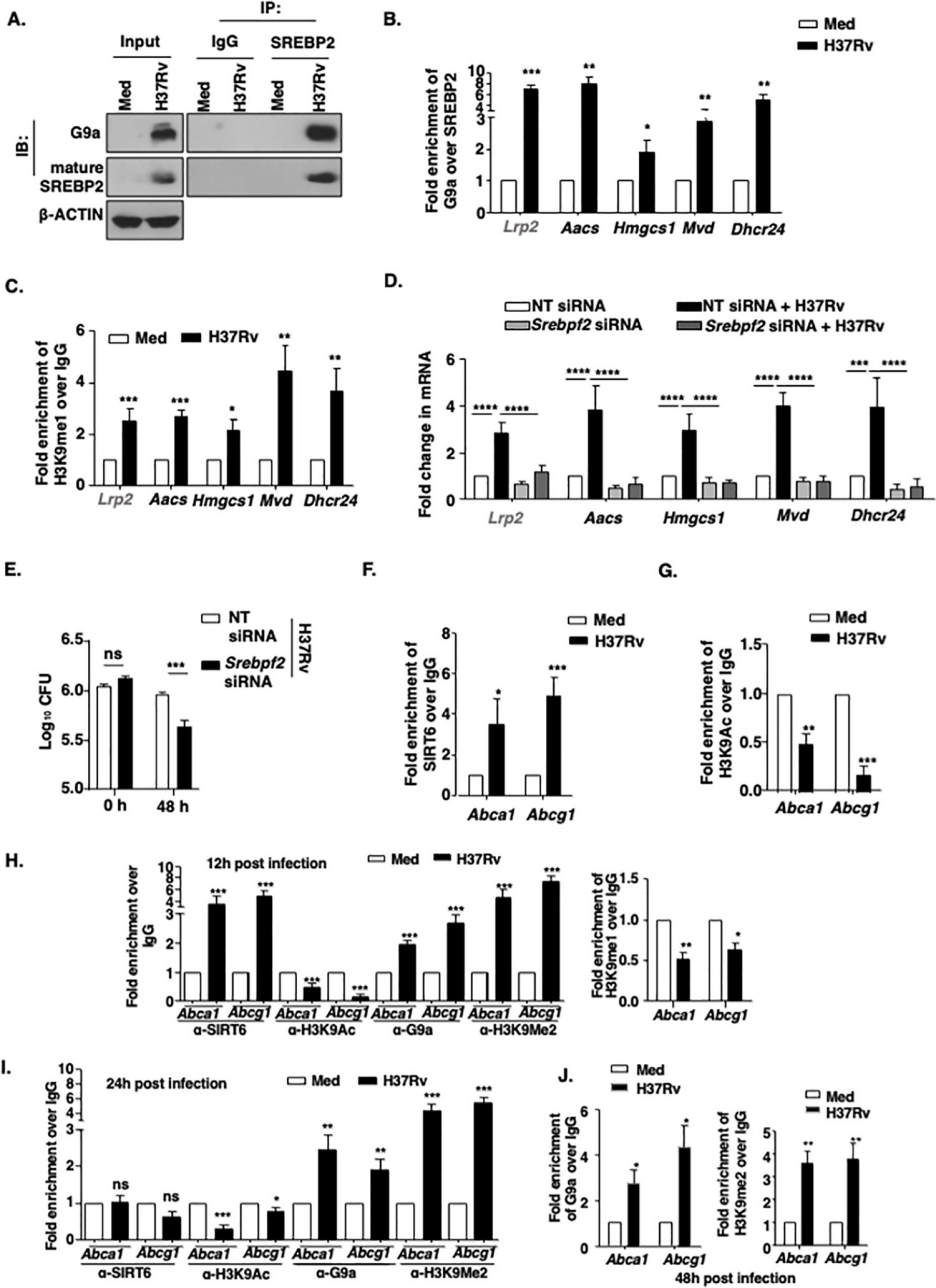
**Mtb induced G9a-SREBP2 and SIRT6 transcriptionally regulate cholesterol biosynthesis and transport genes (A)** SREBP2 was immunoprecipitated in whole cell lysates of BALB/c mouse peritoneal macrophages infected with H37Rv for 12 h to assess its interaction with G9a by immunoblotting. **(B)** Sequential ChIP was performed to assess the co-recruitment of SREBP2 and G9a at the promoters of *Lrp2*, *Aacs*, *Hmgcs1*, *Mvd* and *Dhcr24* in mouse peritoneal macrophages infected with H37Rv for 12 h. **(C)** ChIP assay was performed to affirm the enrichment of H3K9me1 mark, on the promoters of the indicated genes upon 12 h infection with H37Rv in BALB/c mouse peritoneal macrophages. **(D, E)** BALB/c mouse peritoneal macrophages were transfected with *Srebpf2* siRNA and **(D)** infected for 12 h with H37Rv to assess the expression of cholesterol accumulation genes by qRT-PCR, or **(E)** infected with H37Rv for 48 h (MOI 1:5) to analyze the *in vitro* CFU. **(F-G)** BALB/c mouse peritoneal macrophages infected with H37Rv for 12 h were analyzed by ChIP for **(F)** SIRT6 recruitment and **(G)** H3K9Ac mark, on the promoters of *Abca1* and *Abcg1*. **(H)** BALB/c mouse peritoneal macrophages were infected with H37Rv for 12 h, and assessed for the recruitment of SIRT6, G9a and presence of H3K9Ac, H3K9me2 and H3K9me1, on the promoters of Abca1 and Abcg1. **(I)** BALB/c mouse peritoneal macrophages were infected with H37Rv for 24h and assessed for recruitment of SIRT6, G9a, H3K9Ac and H3K9me2 on promoters of Abca1 and Abcg1. **(J)** BALB/c mouse peritoneal macrophages were infected with H37Rv for 48h and assessed for recruitment of G9a and H3K9me2 on promoters of Abca1 and Abcg1.The MOI of infection is 1:10 (macrophage: mycobacteria) for all the *in vitro* experiments. All data represent the mean ± SEM from 3 independent experiments. The blots are representative of 3 independent experiments. *, P < 0.05; **, P < 0.01; ***, P < 0.001; ****, P < 0.0001; ns, not significant (One-way ANOVA for B, D, Student’s t-test for C, E-J). Med, Medium; NT, non-targeting.

In view of above results, we were interested in delineating the G9a- and SIRT6-driven mechanism of differential regulation of cholesterol biosynthesis, uptake, and efflux genes. The transcription factor SREBP2 (encoded by *Srebf2*) is a well-established master regulator of cholesterol biosynthesis genes. However, it functions in tight association with accessory transcription activators and regulators [47]. Towards this end, we observed increased levels of SREBP2 upon Mtb H37Rv infection **(S4A Fig).** We further hypothesized the possibility of SREBP2 and G9a interaction to bring about the augmented expression of cholesterol biosynthesis and uptake genes. Immune-pulldown analysis indicates that SREBP2 interacts with G9a during Mtb H37Rv infection **(Fig 3A)**. To further validate our observation that G9a-SREBP2 activation complex binds to the promoter of cholesterol biosynthesis and uptake genes we performed sequential ChIP which revealed enhanced co-occupancy of the concerned promoters with both G9a and SREBP2 **(Fig 3B)**, thus confirming the concerted binding of SREBP2 and G9a to the promoter of cholesterol biosynthesis and uptake genes (*Lrp2*, *Aacs*, *Hmgcs*, *Mvd*, *Dhcr24*). In line, we also observed enhanced enrichment of H3K9me1 gene activation mark imparted by G9a at the promoters of cholesterol biosynthesis and uptake genes (*Lrp2*, *Aacs*, *Hmgcs*, *Mvd*, *Dhcr24*) **(Fig 3C)**. The loss-of-function of SREBP2 using specific siRNA **(S4B Fig,** knockdown validation**)** compromised the expression of positive factors of cholesterol accumulation (*Lrp2*, *Aacs*, *Hmgcs*, *Mvd*, *Dhcr24*) **(Fig 3D)** with a concomitant decrease in the Mtb H37Rv intracellular burden in macrophages **(Fig 3E)**. Further, SIRT6 was found to occupy the promoters of *Abca1* and *Abcg*1 during Mtb H37Rv infection (**Fig 3F)**, leading to concomitantly decreased H3K9 acetylation marks (**Fig 3G)**; thereby supporting the initially observed downregulation of the cholesterol efflux genes during Mtb infection. Further, since H3K9me2 conferred by G9a renders a closed chromatin state and transcriptional downregulation, the contribution of G9a in the reduced expression of ABCA1 and ABCG1 was analyzed. It was found that mycobacteria lost the ability to downregulate protein levels of ABCA1 in *Ehmt2* knocked down macrophages **(S4C Fig)** and transcript levels of *Abca1* and *Abcg1* **(S4D Fig)**; indicating the partial dependence of cholesterol efflux genes on the repressive function of G9a. Silencing of G9a had no impact on the expression levels of the unaltered cholesterol biosynthesis genes **(S4E Fig)**. Also, silencing of SIRT6 did not have any effect on cholesterol uptake or biosynthesis genes (**S4F Fig**).

We also carried out kinetics study evaluating the recruitment of histone modifiers (G9a and SIRT6) or their associated histone marks (H3K9me and H3K9ac) at the promoters of *Abca1* and *Abcg1*. We performed ChIP assays at various time points ranging from 12 to 48 hours. Our findings demonstrate **(Fig 3H)** that SIRT6 (histone deacetylase) is recruited to the promoters of *Abca1* and *Abcg1* as early as 12 hours post Mtb infection, resulting in a simultaneous decrease in H3K9 acetylation. However, at 24 hours post-infection (hpi) **(Fig 3I),** we observed a loss of SIRT6 recruitment, indicating a potential role of SIRT6 in regulating the expression of *Abca1* and *Abcg1* during the early stages of infection. We also observed an increased recruitment of G9a and H3K9me2 (histone di-methylation) at 12 hpi, which persisted until 48 hpi **(Fig 3J).** Interestingly, the levels of H3K9me were reduced at 48 hpi, suggesting a likely conversion from H3K9 mono methylation to dimethylation mediated by G9a. In summary, our findings suggest that during the early stages of infection, SIRT6 deacetylates H3K9, creating a platform for G9a-mediated demethylation of H3K9, ultimately leading to the transcriptional inactivation of *Abca1* and *Abcg1*.

These sets of analysis show that G9a along with SREBP2 regulated cholesterol accumulation genes (*Lrp2*, *Aacs*, *Hmgcs*, *Mvd*, *Dhcr24*). Whereas SIRT6 and G9a suppress cholesterol efflux genes *Abca1* and *Abcg1*.

To evaluate the interplay between cholesterol accumulation, bacterial burden, and the role of specific genes involved in cholesterol metabolism during Mtb infection, we have carried out in vitro CFU assay upon addition of exogenous cholesterol. We observed supplementation with cholesterol (50μg) was able to induce cholesterol accumulation in macrophages **(S4G Fig)** and was able to rescue bacterial survival in the case of G9a and SIRT6 knockdown, supporting our hypothesis that G9a and SIRT6-mediated increase in cholesterol accumulation creates a more favorable environment for Mtb within the host **(S4H Fig).** Furthermore, we found that cholesterol supplementation had no effect on the reduced bacterial survival in macrophages where cholesterol synthesis and uptake genes were silenced. This aligns with the role of *Lrp2* in cholesterol uptake during Mtb infection, indicating that exogenous supplementation of cholesterol does not impact intracellular Mtb survival in the absence of functional cholesterol uptake. Our cell viability assays showed no change in cell viability during Mtb infection along with siRNA mediated knockdown of cholesterol accumulation genes **(S4I Fig).**

### Cholesterol accumulation mitigates oxidative stress during mycobacterial infection

**Fig 4:**
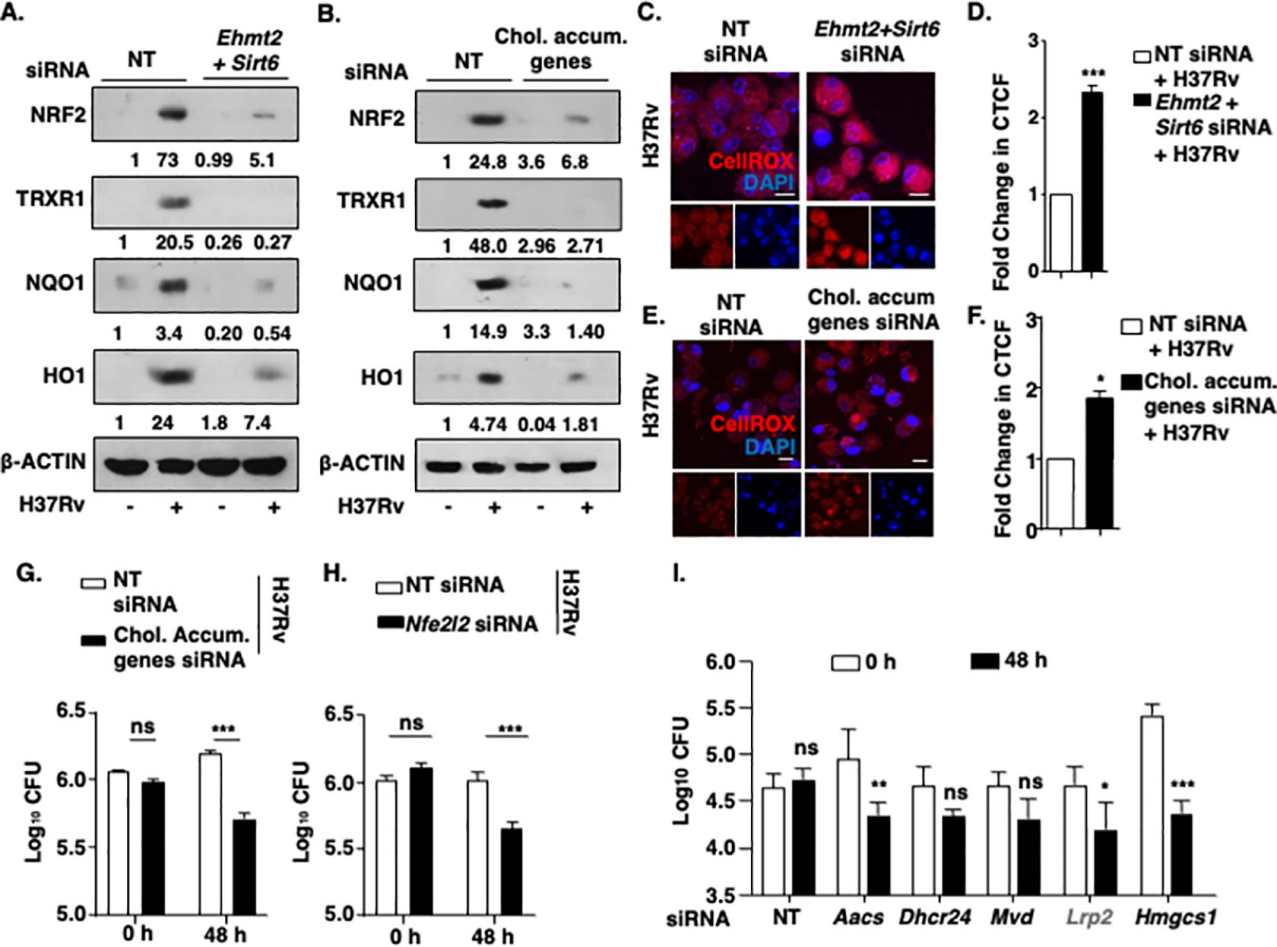
Cholesterol accumulation regulates the expression of antioxidant genes during mycobacterial infection. **(A-I)** BALB/c mouse peritoneal macrophages were transfected with NT or *Ehmt2* and *Sirt6* siRNA or *Nfe2l2* siRNA or cholesterol accumulation genes siRNA (*Lrp2*, *Aacs*, *Hmgcs1*, *Mvd* and *Dhcr24* siRNAs) followed by 48 h of H37Rv infection. **(A, B)** The expression of the indicated molecules was assessed at the protein level by immunoblotting. **(C-F)** CellROX staining was performed to assess ROS levels in macrophages; **(C, E)** representative images and **(D, F)** respective quantification (CTCF normalized to NT, represented as fold change). **(G, H, I)** *In vitro* CFU (MOI 1:5) was assessed. The MOI of infection is 1:10 (macrophage: mycobacteria) for all the *in vitro* experiments unless otherwise stated. All data represent the mean ± SEM from 3 independent experiments. The blots are representative of 3 independent experiments. *, P < 0.05; **, P < 0.01; ***, P < 0.001; ns, not significant (Student’s t-test for D, F, G-I). Med, Medium; NT, non-targeting; chol. accum. genes, cholesterol accumulation genes. Scale bar, 10μm.

The orchestrated accumulation of cholesterol by Mtb-induced G9a and SIRT6 provides insights into the crucial functions that cholesterol might effectuate to favor Mtb survival. As discussed, the contribution of cholesterol as a source of nutrition for mycobacteria is widely accepted. However, evidence from the literature suggests numerous alternate implications of cholesterol in cellular homeostasis and responses to stimuli [48, 49]. To our interest, supplementation of exogenous cholesterol in nonalcoholic steatohepatitis has been shown to help in mitigating the toxic effects of bile acid and lipids by enhancing the expression of NRF2 (Nuclear Factor-Erythroid 2- related factor 2) and HIF-1α (Hypoxia Inducible Factor 1) [50]. NRF2 is one of the key transcription factors that regulates the expression of various anti-oxidative genes in response to oxidative stress [51]. Similarly, cholesterol crystals present in the atherosclerotic plaques have also been reported to act as a stimulus to regulate NRF2 [52]. With this premise, we assessed if the cholesterol accumulated during mycobacterial infection has any effect on the expression of NRF2 and associated anti-oxidative responses. In this regard, we observed that infection with Mtb activates anti-oxidative response by triggering the expression of principal transcription activator of antioxidant genes, NRF2 (encoded by *Nfe2l2*) that then leads to the expression of its target genes **(S5A and S5B Fig)**. The expression of antioxidant genes (*Nqo1*, *Gsr*, *Hmox1*, *Txnrd1*, *Gpx1 and Sod1*) was found to be NRF2-dependent as silencing of NRF2 using specific siRNA **(S5C Fig;** knockdown validation**)** caused significant downregulation of the concerned antioxidant genes **(S5D Fig)**. To assess if the expression of NRF2-dependent antioxidant genes were dependent on G9a- and SIRT6-dependent cholesterol during H37Rv infection, we used specific siRNAs. The expression of NRF2-target genes was found to be compromised both at the transcript **(S5E Fig)** and protein **(Fig 4A)** level upon G9a and SIRT6 silencing. We next verified that the observed loss of antioxidant gene expression indeed resulted from attenuated accumulation of cholesterol in *Ehmt2*- and *Sirt6*-deleted cells. To this end, first we utilized siRNAs against the five G9a-dependent genes found to be essential for cholesterol biosynthesis and uptake (*Lrp2*, *Aacs*, *Hmgcs*, *Mvd*, *Dhcr24*) (**S5F Fig:** Validation of siRNA mediated knock down and **S5G Fig:** cell viability, respectively). In these cholesterol deficient cells, we found a significant reduction in the expression of antioxidant genes at the transcript and protein level (**Fig**s **S5E and 4B**); thus, implicating cholesterol in driving antioxidant gene expression. To directly measure the effect of antioxidant gene alteration we assessed cellular oxidative stress levels using CellROX. In line with literature, peritoneal macrophages showed increased oxidative stress upon Mtb H37Rv infection (**S5H and S5I Fig**) [53]. Interestingly, macrophages silenced for G9a and SIRT6 (**Fig 4C and 4D**) or for G9a-dependent cholesterol biosynthesis/uptake genes (**Fig 4E and 4F**) showed a further increase in oxidative stress upon H37Rv infection. Corollary to this, we also observed that *in vitro* depletion of cholesterol accumulation genes (**Fig 4G**) or *Nfe2l2* (**Fig 4H**) in macrophages significantly compromised Mtb H37Rv burden. Furthermore, to identify the importance of each of the G9a-dependent cholesterol biosynthesis and uptake genes, each gene was individually knocked down and assessed for their effect on the Mtb burden (**Fig 4I**). Our validation experiment confirms the decrease in cholesterol levels upon knockdown of cholesterol biosynthesis and uptake genes (*Lrp2*, *Aacs*, *Hmgcs*, *Mvd*, *Dhcr24*) in 48h Mtb infected peritoneal macrophages **(S5J Fig).** No change in the basal cholesterol levels upon silencing cholesterol biosynthesis and uptake genes in the uninfected scenario (NT siRNA Vs Chol. Accum. Genes siRNA) underscores the role for these genes in facilitating Mtb-mediated accumulation of cholesterol, i.e., the ability of Mtb to induce cholesterol accumulation was perturbed. Our observations suggest a dominant role for the genes *Hmgcs1* and *Aacs*, catalyzing the rate limiting steps of cholesterol biosynthesis, in impacting mycobacterial survival. These observations highlight the critical functions of cholesterol accumulation in mycobacteria-infected hosts.

### G9a- and SIRT6-mediated cholesterol accumulation is regulated by H37Rv driven-WNT pathway

**Fig 5:**
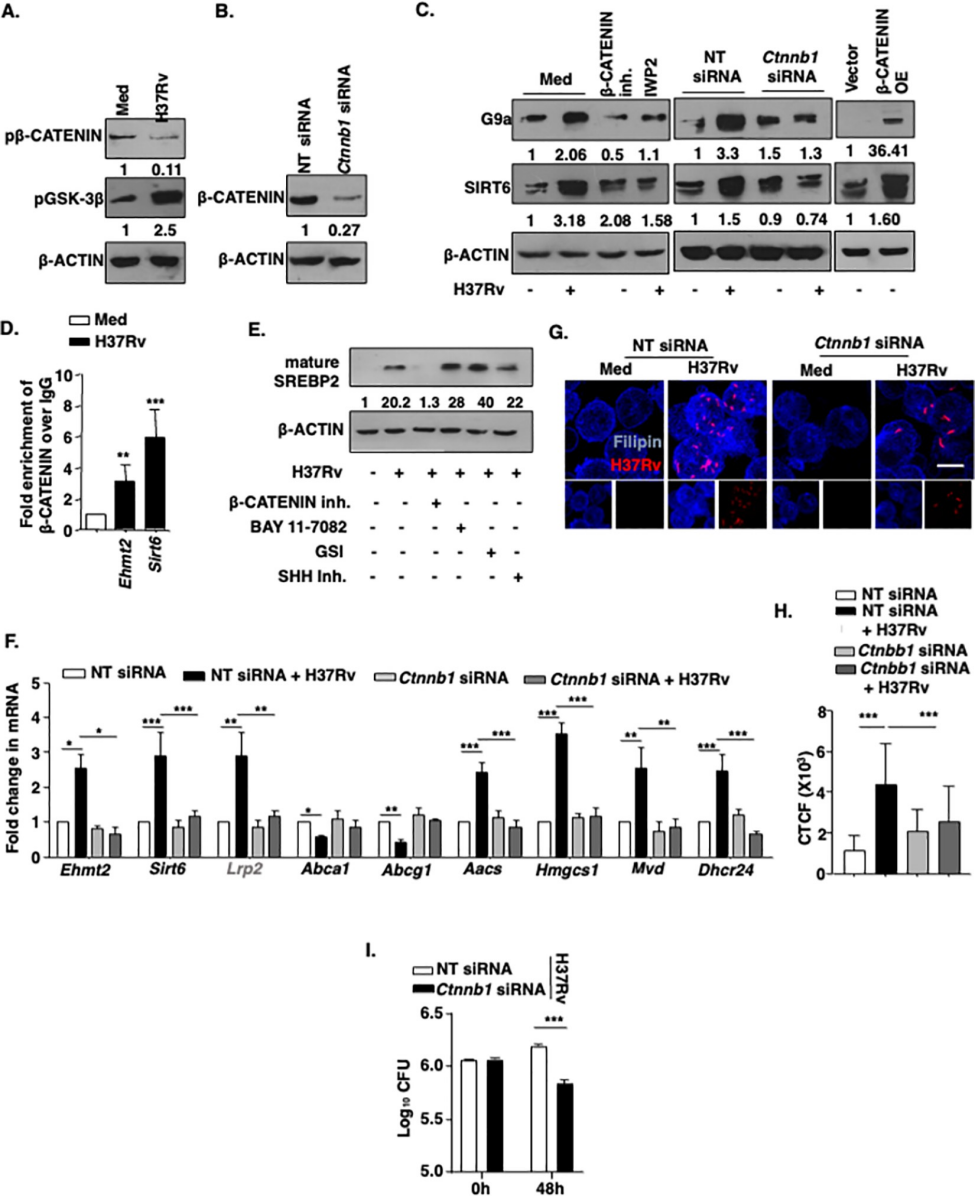
Contribution of WNT/β-CATENIN axis in G9a/SIRT6 induced cholesterol accumulation upon mycobacterial infection. **(A)** BALB/c mouse peritoneal macrophages were infected with H37Rv for 1 h and whole cell lysates were assessed for the activation of WNT pathway. **(B)** Immunoblotting to validate β-CATENIN knockdown in BALB/c mouse macrophages transfected with NT or *Ctnnb1* siRNA. **(C)** RAW 264.7 macrophages were transfected with β-CATENIN OE construct (**C**, left panel) or mouse peritoneal macrophages were transfected with NT or *Ctnnb1* siRNA (**C**, middle panel) or BALB/c mouse peritoneal macrophages were pre-treated with β-CATENIN inhibitor (15 μM) or IWP2 (5 μM) for 1 h (**C**, right panel), followed by 12 h infection with H37Rv. Whole cell lysates were assessed for SIRT6 and G9a expression by immunoblotting. **(D)** β-CATENIN recruitment at the promoter of *Ehmt2* and *Sirt6* was assessed by ChIP assay in BALB/c mouse primary macrophages infected with H37Rv for 12 h. **(E)** BALB/c mouse peritoneal macrophages were infected with H37Rv for 12 h in presence and absence of the indicated inhibitors and whole cell lysates were assessed for mSREBP2. **(F)** Indicated genes were analyzed at transcript level by qRT- PCR in BALB/c mouse peritoneal macrophages that were transfected with NT or *Ctnnb1* siRNA followed by infection with H37Rv for 12 h. **(G, H)** Free cholesterol was assessed by Filipin staining in BALB/c mouse peritoneal macrophages transfected with NT or *Ctnnb1* siRNA followed by 48 h infection with tdTomato H37Rv, **(G)** representative image and **(H)** respective quantification. **(I)** BALB/c mouse macrophages were transfected with NT or *Ctnnb1* siRNA and *in vitro* CFU was assessed at the indicated time points post H37Rv (MOI 1:5) infection. The MOI of infection is 1:10 (macrophage: mycobacteria) for all the *in vitro* experiments unless otherwise stated. All data represents the mean ± SEM from 3 independent experiments, *, P < 0.05; **, P < 0.01; ***, P < 0.001 (Student’s t- test for D and I; One-way ANOVA for F & H). All blots are representative of 3 independent experiments. Med, medium; β-CATENIN OE, β-CATENIN overexpression; NT, non-targeting; inh., inhibitor. Scale bar, 10 μm.

Mycobacterial infection has been shown to activate various developmental pathways like WNT, SHH, NOTCH, Hippo [45, 54–56] pathways in mouse macrophages. Importantly, the WNT pathway has been associated with antioxidant genes master regulator NRF2 for defining neuronal developmental pathways [57]. Activated WNT signaling, driven by WNT3A, has also been shown to enhance NRF2-mediated antioxidant gene expression by preventing the GSK3β-dependent phosphorylation and subsequent proteasomal degradation of NRF2 in hepatocytes [58]. Importantly, WNT ligand WNT6 has been implicated in foam cell formation during pulmonary TB [59]. Further, its contribution in regulating lipid accumulation by endocytosis of LDL-derived cholesterol [60] indicated its possible role in yet another aspect of Mtb infection, i.e., cholesterol accumulation. In the perspective of the above-mentioned observations, we explored the role of the WNT signaling pathway in controlling Mtb-driven expression of G9a and SIRT6. The WNT pathway was activated in mouse macrophages upon Mtb H37Rv infection as seen by increased pGSK3β and reduced pβ-CATENIN levels **(Fig 5A)**. Importantly, G9a and SIRT6 expression was found to be dependent on Mtb H37Rv-activated WNT pathway as inhibition of the pathway with pharmacological inhibitors (IWP2 and β-CATENIN inhibitor) (**Fig 5C, left panel**) or knockdown of *Ctnnb1* (**Fig 5C, middle panel; Fig 5B:** knockdown validation) compromised the levels of G9a and SIRT6. Conversely, β-CATENIN overexpression alone induced the expression of the concerned histone modifiers independent of Mtb infection (**Fig 5C, right panel**). Further, β-CATENIN was found to be recruited to the promoters of *Ehmt2* and *Sirt6*, (**Fig 5D**). By using a panel of inhibitors for various signaling pathways like WNT, NF-κB, NOTCH and SHH, SREBP2 expression was found to be specifically regulated by the WNT pathway (**Fig 5E**). In line, siRNA-mediated knockdown of *Ctnnb1* compromised the ability of Mtb to differentially regulate cholesterol metabolism genes (**Fig 5F**); subsequently cholesterol accumulation (**Fig 5G and 5H)** and hence Mtb H37Rv survival (**Fig 5I**). These findings indicate that Mtb infection leads to the WNT signaling pathway-dependent expression of G9a/SIRT6 as well as accumulation of cholesterol, which drives a secure niche for the pathogen to survive.

### G9a and SIRT6 contribute to mycobacterial pathogenesis

**Fig 6:**
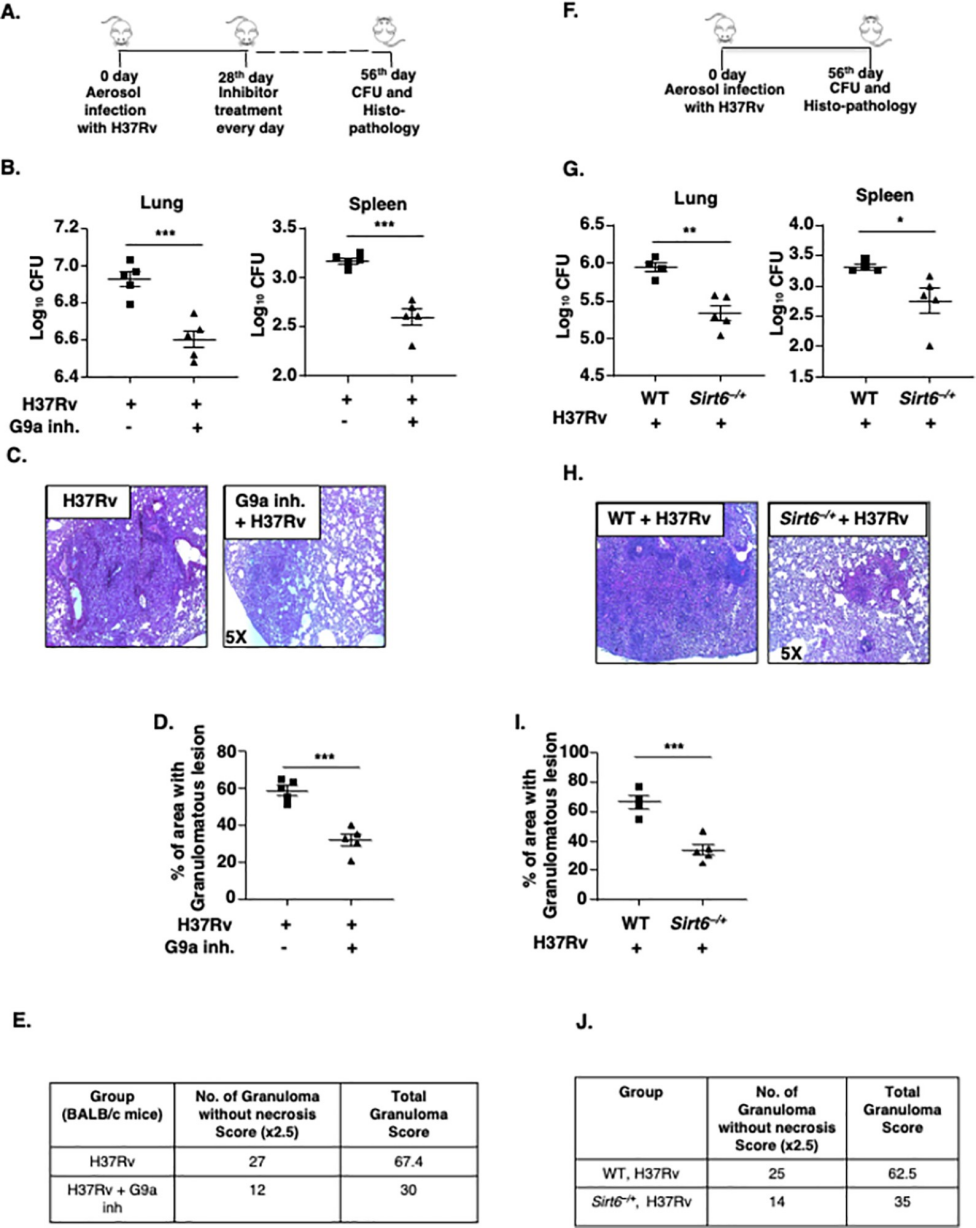
Epigenetic modifiers G9a and SIRT6 aid in mycobacterial pathogenesis. **(A-J)** Mice were aerosolized with 200 CFU of H37Rv. **(A)** Schematic of *in vivo* mouse TB model for G9a inhibitor therapeutic treatment. **(B)** H37Rv CFU from the lungs and spleen of G9a inhibitor (40mg/kg) treated and untreated BALB/c mice was assessed after 56 days of total infection and therapeutic treatment. **(C-D)** Lungs of BALB/c mice from the indicated groups were analyzed for TB pathology by H & E staining; **(C)** representative image, **(D)** % of granulomatous area and **(E)** corresponding histological evaluation for granuloma score. **(F)** Schematic of *in vivo* Mtb infection model for WT (littermate control) and *Sirt6*^*−/+*^ mice. **(G)** CFU of H37Rv from the lung and spleen of WT (littermate control) and *Sirt6*^*−/+*^ mice were assessed following 56 days of H37Rv infection. **(H, I)** Lungs from the indicated groups of mice were analyzed by H and E staining; **(H)** representative images, **(I)** % of granulomatous area and **(J)** corresponding histological evaluation for granuloma score. N = 2 for Fig 6A, B; N = 1 for Fig 6C–6J. All data represents the mean ± SEM from 4–5 mice, *, P < 0.05; **, P < 0.01; ***, P < 0.001 (Student’s *t*-test for B, D, G and I). WT, Wild type; inh., inhibitor.

The observed G9a/SIRT6-dependent accumulation of cholesterol and the related abatement of mycobacterial burden upon their functional loss incited us to determine the impact of G9a and SIRT6 in defining *in vivo* Mtb burden and associated lung tissue pathology during Mtb H37Rv infection. We found that therapeutic treatment of Mtb H37Rv-infected mice with G9a inhibitor **(Fig 6A)** not only compromised cholesterol accumulation but also reduced mycobacterial CFU **(Fig 6B)** and led to a decreased level of Mtb infection-specific granuloma-like lesions **(Fig 6C)**. Lung histopathological examination by Hematoxylin and Eosin (H and E) staining also revealed a marked reduction in the percentage of lung area covered with the characteristic TB granuloma-like lesions **(Fig 6D)**, with an overall decline in total granuloma score compared to the untreated counterparts (**Fig 6E and S6A Fig**). Further, we observed reduced Mtb H37Rv CFU in the lungs and spleen of *Sirt6*^*−/+*^ mice **(Fig 6F and 6G)** and up to 50% restriction in the ability of *Sirt6*^*−/+*^ mice to effectively develop TB granuloma-like lesions (**Fig 6H–6J and S6B Fig**). Therefore, the significant normalization of total lung architecture in mice lacking G9a or SIRT6 functions strongly indicates the relevance of the histone modifications conferred by G9a and SIRT6 in the pathogenesis of Mtb. We believe that thwarted cholesterol accumulation, leading to enhanced oxidative stress, jeopardizes mycobacterial survival strategies, thereby restricting overall TB progression in mice with abrogated G9a/SIRT6 functions.

## Discussion

The formation of FMs has been described as an integral part of TB pathogenesis and the constituents of the lipid droplets (LDs) contained in the FMs are associated with diverse functions. Specifically, cholesterol uptake by Mtb and utilization to achieve survival advantages has been vividly elucidated [61–64]. We uncover the Mtb-driven host molecular players that lead to the accumulation of this essential factor in host cells during infection. Despite the presence of compelling evidence for the implication of cholesterol in the pathogenesis of Mtb, the epidemiological surveys depict a nonlinear and complex relationship between high cholesterol and TB progression [65]. Similarly, in mouse models of pulmonary TB, a cholesterol-rich diet (high serum cholesterol levels) has been related to distinct disease outcomes. For instance, in *Apoe*^−/−^ mice, high serum cholesterol impairs host defense against Mtb [66]; while that in *Ldlr*^−/−^ mice does not alter the capacity of the host to restrict mycobacterial replication [67]. These differences may be explained by the differences in cholesterol availability that arise from its esterification or association with lipoproteins to form VLDLs, LDLs and HDLs. Therefore, a clear picture defining the role of cholesterol still warrants investigation.

The accumulation of cholesterol imparts regulatory effects on several aspects of host immunity by altering processes ranging from plasma membrane dynamics to maintaining serum cholesterol levels and epigenetic deregulations. Cholesterol is important for the adaptive immune system for its contribution to the formation of plasma membrane lipid rafts, which facilitate immune functions such as T-cell and B-cell signaling, their activation and proliferation [68][69]. Further, high serum cholesterol leads to autoimmune and inflammatory manifestations via aberrant immune activation [70]. Alongside these important roles, cholesterol accumulation also shapes the innate immune arm by modulating functions such as TLR signaling, monocyte proliferation, macrophage polarization, apoptosis as well as dendritic cell maturation and activation under distinct conditions [71–75], including infections. For instance, cholesterol has been shown to play a crucial role in regulating Salmonella-induced autophagy [76] and lowering free cholesterol by their conversion to oxysterols has been implicated in providing immunity against *Listeria monocytogenes* infection [77].

During mycobacterial infection suppression of intracellular cholesterol accumulation via oxysterols (natural LXR activators) or by inhibition of SREBP2 has been shown to enhance the production of antimicrobial peptides that restrict Mtb burden [78]. In line, loss of function of LXRα and LXRβ (leading to reduced expression of *Abca1*) has been reported to render mice more susceptible to Mtb infection due to defective recruitment of innate effector cells and innate immune functions as well as severely compromise Th1/Th17 functions [79]. It should be noted that literature suggests that SIRT6 inhibits SREBP2 expression [37]. Also, SIRT6 induces the expression of *Abca1* and *Abcg1* [80] in the premise of oxidized LDL. However, we must appreciate the ability of Mtb to modulate multiple pathways in host cells. For example, in contrast to reduced miR33 expression found in ox-LDL exposed SIRT6 overexpressing cells [80], Mtb infection has been reported to induce miR33 expression in host cells [18]. Further, papers show the ability of G9a to repress adipogenesis, however it is in the context of its repressive H3K9me2 activity on the promoters of PPARγ [81]. Whereas, in the event of Mtb infection, PPARγ activity has been reported to be enhanced [82], thereby leading us to speculate the role for Mtb-induced G9a that is distinct from its PPARγ -inhibitory activity. In the current study, we present a novel mechanism by which free cholesterol that accumulates within host cells can aid in Mtb pathogenesis. We show that G9a-SREBP2 and SIRT6 independently regulates the cholesterol homeostasis wherein G9a-SREBP2 upregulates cholesterol biosynthesis genes and SIRT6 downregulates the expression of cholesterol efflux genes without any effect on the cholesterol biosynthesis genes. Mechanistically, we found SREBP2 to be specifically under the control of WNT signaling and underscored its role in regulating G9a and SIRT6 as well as cholesterol synthesis and uptake genes during Mtb infection. Other factors could be involved in the regulation of G9a and SIRT6, however, we underpin a possible role of WNT pathway in mediating cholesterol accumulation via regulating these epigenetic factors during TB. We find that cholesterol accumulation modulates the innate immune arm by driving the expression of anti-oxidative genes that would favor Mtb survival by circumventing oxidative stress responses and mediators. This aligns with the observation that cells with high cholesterol upregulate antioxidants such as NRF2 and HO-1 to mitigate oxidative stress [83]. A recent report from our lab proposes that mycobacterial clearance pathways such as apoptosis and pro-inflammatory cytokine production are hampered by classical anti-oxidative molecules TRXR1 and NQO1 [5]. Therefore, cholesterol-dependent antioxidant production and subsequent innate and adaptive immune alterations not reported as yet, can potentially help in strengthening the understanding of the survival strategies employed by Mtb.

In the light of host-directed therapeutics, our finding is in congruence with a previous study where statins, that decrease cholesterol levels by inhibiting HMGCoA reductase (a rate-limiting step of cholesterol biosynthesis), had been reported to inhibit mycobacterial growth [84]. With the individual knockdown of G9a-dependent cholesterol biosynthesis genes, we tease out the specific contribution of *Hmgcs1* and *Aacs* in regulating cholesterol-driven mycobacterial burden. Therefore, this study provides an avenue for testing alternate targets for effective combinatorial therapy against TB and for dedicated studies on metabolic homeostasis and mycobacterial pathogenesis in *Hmgcs1* or *Aacs* knockout conditions. Recently, mammalian sirtuins have been proposed as a potential target for host-directed therapy against TB. For example, SIRT1 activators ameliorates lung pathology, SIRT3 promotes antimycobacterial responses whereas SIRT2 inhibition has been shown to reduce Mtb burden [85] [24, 26]. In the current study, we find that SIRT6 benefits the Mtb survival and impacts lung pathology, thereby establishing the class of sirtuins as potential targets for TB therapeutics.

**Fig 7:**
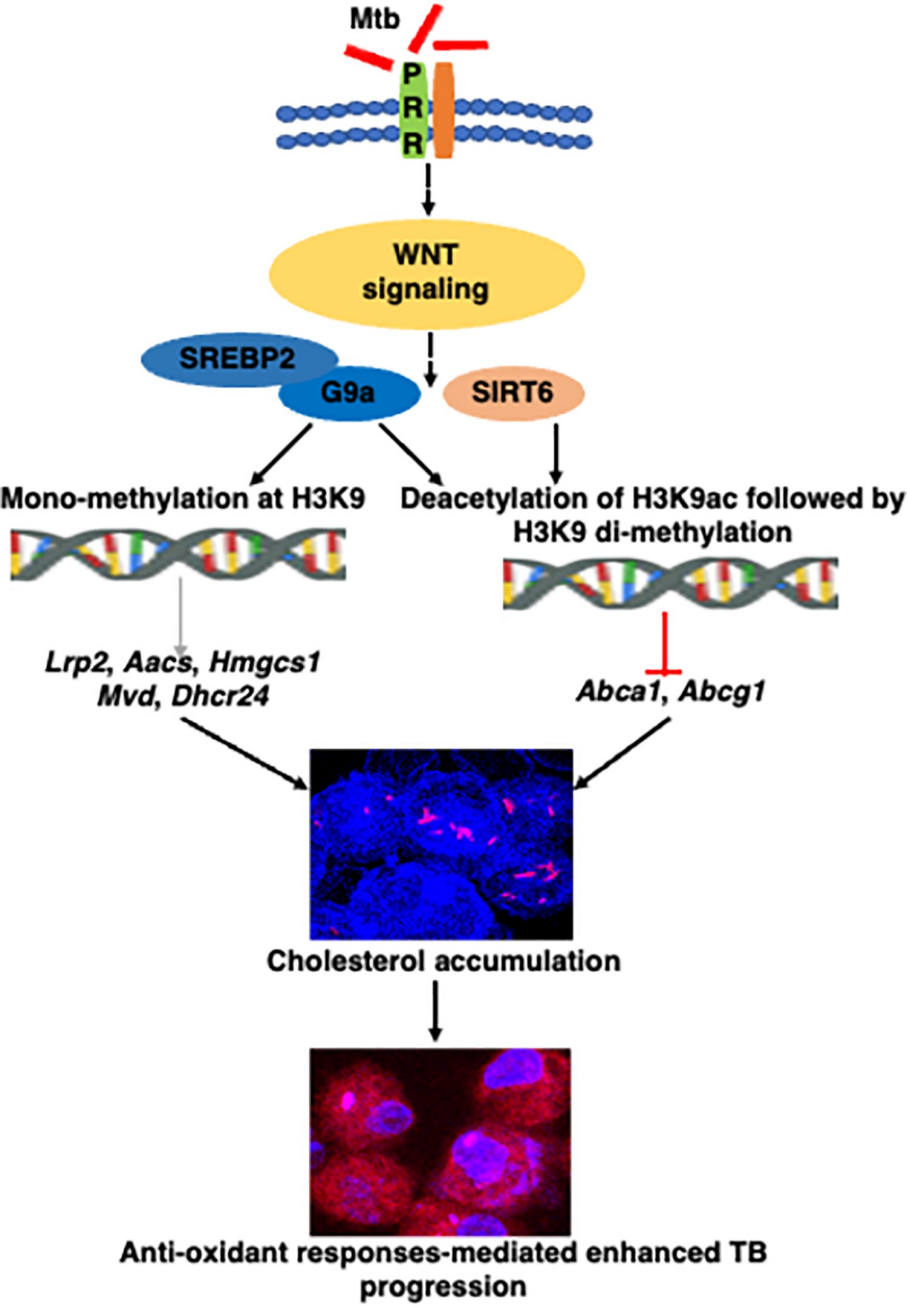
Schematic, Mycobacteria utilizes the WNT signaling-dependent host epigenetic factors G9a and SIRT6 to augment cholesterol accumulation and antioxidant responses in order to aid its survival within the host.

Together, we report that epigenetic modifiers G9a and SIRT6 are induced by Mtb, that differentially regulates the expression of cholesterol biosynthesis, uptake, and efflux genes, in order to build up cholesterol within host cells. Interception of G9a and SIRT6 restricts mycobacterial burden and limits TB-like pathology, plausibly by compromising free cholesterol accumulation and thereby increasing oxidative stress in host cells **(Fig 7)**. We believe that an organ-specific and carefully titrated delivery of therapeutics against these epigenetic factors would provide rational and clinically relevant adjuvants for TB treatment.

## Materials and methods

### Ethics statement

Experiments involving mice and virulent mycobacteria (Mtb H37Rv) were carried out after the approval was granted from Institutional Ethics Committee for animal experimentation and Institutional Biosafety Committee. The animal care and use protocol adhered were granted approval by national guidelines of the Committee for Control and Supervision of Experiments on Animals (CCSEA) (formerly CPCSEA), Government of India. Experiments involving human samples (PBMCs) were carried out after approval was granted from the Institutional Human Ethics Committee (IHEC).

### Mice and cells

Male and female mice of the following strains were utilized in the study: BALB/c (stock number 000651, The Jackson Laboratory, USA), *Sirt6*^−/−^ (kind gift from Dr. Ullas Kolthur-Seetharam, TIFR, India and Dr. Nagalingam Ravi Sundaresan, IISc, India; primary source: The Jackson Laboratory, USA, stock number 006050) and *Sirt6*^*−/+*^ (kind gift from Dr. Ullas Kolthur-Seetharam, TIFR, India and Dr. Nagalingam Ravi Sundaresan, IISc, India; primarily generated by crossing *Sirt6*^−/−^ mice with WT 129S6 mice). Mouse primary macrophages were isolated from peritoneal exudates using ice-cold PBS four days post intraperitoneal injection of 1.5ml of brewer thioglycollate (8%). RAW 264.7 mouse macrophages cell line was obtained from ATCC and National Center for Cell Sciences, Pune, India; and used for transient transfection experiments using plasmids as they are better suited for transfection as compared to the peritoneal macrophages that are known to be highly sensitive to external DNA [86][87]. Primary macrophage and RAW 264.7 macrophage cell line was cultured in Dulbecco’s Minimal Eagle Medium (Gibco, Thermo Fisher Scientific) supplemented with 10% heat-inactivated Fetal Bovine Serum (Gibco, Thermo Fisher Scientific) and maintained at 37°C in 5% CO_2_ incubator. Cell line used was tested mycoplasma negative by PCR based assay. All strains of mice were obtained from The Jackson Laboratory and maintained in the Central Animal Facility (CAF), Indian Institute of Science (IISc) under 12 h light and dark cycle.

### Bacteria

Mtb H37Rv was a kind research gift from Prof. Kanury Venkata Subba Rao, THSTI, India. tdTomato Mtb H37Rv was a kind research gift from Dr. Amit Singh, IISc, India. Mycobacterial cultures were grown to mid-log phase in Middlebrook 7H9 medium (Difco, USA) supplemented with 10% OADC (oleic acid, albumin, dextrose, catalase) and hygromycin for tdTomato Mtb H37Rv. Single-cell suspensions of mycobacteria were obtained and used at a multiplicity of infection 10 unless mentioned otherwise. The studies involving virulent mycobacterial strains were carried out at the biosafety level 3 (BSL-3) facility at CIDR, IISc.

### Transient transfection studies

RAW 264.7 macrophages were transiently transfected with 5μg of overexpression constructs of β-CATENIN and SIRT6; or peritoneal macrophages were transfected with 100 nM each of siGLO Lamin A/C, non-targeting siRNA or specific siRNAs against *Ehmt2*, *Sirt6*, *Ctnnb1*, *Lrp2*, *Aacs*, *Hmgcs1*, *Mvd*, *Dhcr24*, *Srebf2*, *Nfe2l2* (purchased from Dharmacon) with polyethyleneimine. 70–80% transfection efficiency was observed by counting the number of siGLO Lamin A/C positive cells in a microscopic field using fluorescence microscopy. 36 h post-transfection (for experiments with RAW 264.7 cells) or 24h post-transfection (for experiments with peritoneal macrophages), the cells were treated or infected as indicated and processed for analyses.

### *In vivo* mouse model and inhibitor treatment

BALB/c mice (n = 40) were infected with mid-log phase Mtb H37Rv, using a Madison chamber aerosol generation instrument calibrated to 200 CFU/animal. Aerosolized animals were maintained in a securely commissioned BSL3 facility. Post 28 days of established infection, mice were administered a daily dose of G9a inhibitor BIX-01294 (40mg/kg) [88] intra-peritoneally for 28 days. Alternately, wild type (littermate control) mice or *Sirt6*^*−/+*^ mice were infected as described above. In each case, on the 56^th^ day, mice were sacrificed, spleen and left lung lobe and spleen were homogenized in sterile PBS, serially diluted and plated on 7H11 agar containing OADC to quantify CFU. Upper right lung lobes were fixed in formalin, embedded in paraffin and stained with hematoxylin and eosin and immunofluorescence analysis. For Granuloma scoring, different scores were assigned based on the characteristic granulomatous features that is granuloma with necrosis (Score = 5), without necrosis (Score = 2.5) and with fibrosis (Score = 1) [89]. For total granuloma scoring, the number of granulomas in each lung lobe was multiplied with the characterized feature score. The granulomatous area of lung sections stained with H&E was measured using Image J software (granulomatous area/ total area *100). in vivo experimentation has been carried out twice (once in male and once in female mice).

### Antibodies

HRP-conjugated anti-β-ACTIN antibody, Filipin and 4′,6-Diamidino-2-phenylindole dihydrochloride (DAPI) were purchased from Sigma-Aldrich/ Merck Millipore. Alexa488-conjugated anti-rabbit IgG, HRP-conjugated anti-rabbit total IgG and light chain specific IgG antibodies were purchased from Jackson ImmunoResearch, USA; PE-conjugated F4/80 was procured from Tonbo Biosciences, USA. Alexa Fluor 660 conjugated CD68 was purchased from ThermoFischer Scientific. Anti-G9a, anti-SIRT6, anti-H3K9me1, anti-H3K9me2, anti-H3K9Ac, anti-Ser33/37/Thr41 phospho-β-CATENIN, anti-Ser9 phospho-GSK-3β, anti-β-CATENIN, anti-NRF2, anti-HO1 and anti-TRXR1 antibodies were obtained from Cell Signaling Technology, USA. Anti-LRP2 antibody was purchased from Santa Cruz Biotechnology, USA; anti-SREBP2 antibody was procured from Abcam, USA; and anti-NQO1 antibody was purchased from Calbiochem, USA.

### Treatment with pharmacological reagents

Cells were treated with concerned pharmacological inhibitors for 1 h prior to the experiment at the following final concentrations: BIX-01294 (G9a inhibitor, 5 μM), β-CATENIN inhibitor (15 μM), IWP-2 (5 μM), BAY 11–7082 (20 μM), NOTCH pathway inhibitor, GSI (10 μM), SHH Inhibitor, Cyclopamine (10 μM). DMSO at 0.1% concentration was used as the vehicle control. In all experiments involving pharmacological reagents, a tested concentration was used after careful titration experiments assessing the viability of the macrophages using the MTT (3-(4,5-Dimethylthiazol-2-yl)-2,5-diphenyltetrazolium bromide) assay.

### MTT assay

siRNA transfected mouse peritoneal macrophages were treated with 3-(4, 5-Dimethylthiazol-2-yl)-2, 5-diphenyltetrazolium bromide (MTT) for 4 h at a final concentration of 0.5 mg/ml. Media was gently removed post incubation and 200 μL of DMSO was added. This solubilized purple formazan crystals were quantified by measuring absorbance at 550 nm in a 96-well plate reader. Viability of siRNA transfected macrophages were assessed relative to non-transfected macrophages.

### Isolation of Human PBMCs

Histopaque-1077 (Sigma-Aldrich, USA) polysucrose solution was utilized to isolate PBMCs from whole blood as per manufacturer’s instruction. Briefly, 3 ml of whole blood was carefully layered onto 3 ml of Histopaque-1077 in a 15 ml conical centrifuge tube followed by centrifugation at 400 × g for 30 min at room temperature. Upper layer was carefully removed without disturbing the opaque interface of mononuclear cells. The interface was transferred into a fresh 15 ml conical centrifuge tube and resuspended in 10 ml isotonic phosphate buffered saline solution. The solution was centrifuged at 250 × g for 10 min, cell pellet was resuspended and cultured in RPMI supplemented with 10% heat inactivated FBS (Gibco-Life Technologies) in the presence of 10 ng/ml M-CSF (PeproTech, USA) for 5 days at 37°C in 5% CO_2_ incubator and utilized for experiments.

### RNA isolation and Real-Time qRT-PCR

Total RNA from treated, untreated and infected macrophages were isolated using TRI reagent (Sigma). 2 μg of total RNA was converted into cDNA using First Strand cDNA synthesis kit (Applied Biological Materials Inc.). Target gene expression was assessed by Real-Time quantitative Reverse Transcription-PCR (qRT-PCR) using SYBR Green PCR mix (Thermo Fisher Scientific). All the experiments were repeated at least 3 times independently to ensure the reproducibility of the results. *Gapdh* was used as internal control. The list of primers is detailed in S1 File.

### Immunoblotting analysis

Cells post treatment and/or infection were washed with 1X PBS. Whole cell lysate was prepared by lysing in RIPA buffer [50 mM Tris-HCl (pH 7.4), 1% NP-40, 0.25% sodium deoxycholate, 150 mM NaCl, 1 mM EDTA, 1 mM PMSF, 1 μg/ml each of aprotinin, leupeptin, pepstatin, 1 mM Na_3_VO_4_, 1 mM NaF] on ice for 30 min. Total protein from whole cell lysates was estimated by Bradford reagent. Equal amount of protein from each cell lysate was resolved on 12% SDS-PAGE and transferred onto PVDF membranes (Millipore) by semi-dry immunoblotting method (Bio-Rad). 5% non-fat dry milk powder in TBST [20 mM Tris-HCl (pH 7.4), 137 mM NaCl, and 0.1% Tween 20] was used for blocking nonspecific binding for 60 min. After washing with TBST, the blots were incubated overnight at 4°C with primary antibody diluted in TBST with 5% BSA. After washing with TBST, blots were incubated with secondary antibody conjugated to HRP for 4h at 4°C. The immunoblots were developed with enhanced chemiluminescence detection system (PerkinElmer) as per manufacturer’s instructions. β-ACTIN was used as loading control.

### Immunoprecipitation assay

Immunoprecipitation assays were carried out following a modified version of the protocol provided by Millipore, USA. In brief, macrophages were gently resuspended and lysed in an ice-cold RIPA buffer. The cell lysates obtained were subjected to pre-clearing with BSA-blocked Protein A beads. The amount of protein was estimated in the supernatant and equal amount of protein was incubated with IgG or anti-SREBP2 antibody for 4 h at 4°C. The immune complexes were captured on protein A agarose beads (Bangalore Genei, India) at 4°C for 4 h. The beads were separated, washed and boiled in the Laemmli buffer for 10 min. These bead free samples were analyzed for respective target molecules by immunoblotting. Light chain specific secondary antibody was used for immunoblotting after immunoprecipitation.

### Chromatin Immunoprecipitation (ChIP) assay

ChIP assays were carried out using a protocol provided by Upstate Biotechnology and Sigma-Aldrich with certain modifications. Briefly, macrophages were fixed with 3.6% formaldehyde for 15 min at room temperature followed by inactivation of formaldehyde with addition of 125 mM glycine for 10 min. Nuclei were lysed in 0.1% SDS lysis buffer [50 mM Tris-HCl (pH 8.0), 200 mM NaCl, 10 mM HEPES (pH 6.5), 0.1% SDS, 10 mM EDTA, 0.5 mM EGTA, 1 mM PMSF, 1 μg/ml of each aprotinin, leupeptin, pepstatin, 1 mM Na_3_VO_4_ and 1 mM NaF]. Chromatin was sheared using Bioruptor Plus (Diagenode, Belgium) at high power for 70 rounds of 30 sec pulse ON and 45 sec pulse OFF. Chromatin extracts containing DNA fragments with an average size of 500 bp were immunoprecipitated with SIRT6 or G9a or H3K9Ac or H3K9me1 or H3K9me2 or β-CATENIN antibodies or rabbit preimmune sera complexed with Protein A agarose beads (Bangalore Genei, India). Immunoprecipitated complexes were sequentially washed with Wash Buffer A, B and TE [Wash Buffer A: 50 mM Tris-HCl (pH 8.0), 500 mM NaCl, 1 mM EDTA, 1% Triton X-100, 0.1% Sodium deoxycholate, 0.1% SDS and protease/phosphatase inhibitors; Wash Buffer B: 50 mM Tris-HCl (pH 8.0), 1 mM EDTA, 250 mM LiCl, 0.5% NP-40, 0.5% Sodium deoxycholate and protease/phosphatase inhibitors; TE: 10 mM Tris-HCl (pH 8.0), 1 mM EDTA] and eluted in elution buffer [1% SDS, 0.1 M NaHCO3]. After treating the eluted samples with RNase A and Proteinase K, DNA was precipitated using phenol-chloroform-ethanol method. Purified DNA was analyzed by quantitative real time RT-PCR. All values in the test samples were normalized to amplification of the specific gene in Input and IgG pull down and represented as fold change in modification or enrichment. All ChIP experiments were repeated at least three times. The list of primers is detailed inS1C File.

### Sequential ChIP assay

The protocol for sequential ChIP was adopted from [90][91]. Briefly, the DNA fragments obtained following sonication [in lysis buffer; 1% SDS, 10 mM EDTA, 50 mM Tris-HCl (pH 8.0)] were immunoprecipitated with SREBP2-complexed Protein A beads. After first pull down, beads were washed with Re-ChIP Buffer [2 mM EDTA, 500 mM NaCl, 0.1% SDS, 1% NP40], followed by elution of DNA in Re-ChIP elution buffer [2% SDS, 15 mM DTT in TE] at 37°C for 30 min. The eluted DNA was subjected to subsequent round to immunoprecipitation with Protein-A beads pre-complexed with G9a or rabbit pre-immune sera. Immunoprecipitated complexes were sequentially washed with Wash Buffer A, B and TE [Wash Buffer A: 20 mM Tris-HCl (pH 8.0), 150 mM NaCl, 2 mM EDTA, 1% Triton X-100, 0.1% SDS and protease/phosphatase inhibitors; Wash Buffer B: 20 mM Tris-HCl (pH 8.0), 2 mM EDTA, 500 mM NaCl, 1% Triton X-100, 0.1% SDS and protease/phosphatase inhibitors; Wash Buffer C: 10 mM Tris-HCl (pH 8.0), 1 mM EDTA, 1% sodium deoxycholate, 1% NP40, 250 mM LiCl and protease/phosphatase inhibitors; TE: 10 mM Tris-HCl (pH 8.0), 1 mM EDTA and protease/phosphatase inhibitors] and eluted [0.1 M NaHCO_3_, 1% SDS], purified and subjected to qRT-PCR (as described previously). The fold change of SREBP2-G9a versus SREBP2-IgG upon infection signified the co-occupancy of the two factors at concerned promoters. The list of primers is given in S1C File.

### Isolation and culture of murine bone marrow derived macrophages

Mice tibia and femur were flushed with ice-cold DMEM containing 10% fetal bovine serum from WT (littermate control) and *Sirt6*^−/−^ mice. Bone marrow was collected in 50 ml tube and bone marrow clusters were disintegrated by vigorous pipetting. The cell suspension was centrifuged at 1500 rpm for 5 min at 4°C followed by two washes with DMEM containing 10% fetal bovine serum. Then the cells were suspended in DMEM containing 10% fetal bovine serum and 20% of L929 cell supernatant and seeded at 1 million cells per well and incubated at 37°C, 5% CO2 and 95% humidity in a CO_2_ incubator. The medium was supplemented on the 3^rd^ and 5^th^ day with DMEM containing 10% fetal bovine serum and 20% L929 cell supernatant. Post 7 days of differentiation, the cells were used for further experiments.

### *In vitro* Mtb CFU

BALB/c peritoneal macrophages transfected with *Ehmt2*, *Sirt6*, Srebf2, cholesterol genes (*Lrp2*, *Mvd*, *Aacs*, *Hmgcs*, *Dhcr24*), *Nfe2l2*, *Ctnnb1* or non-targeting siRNA for 24 h; or BMDMs obtained from *Sirt6*^−/−^ mice were infected with Mtb H37Rv at MOI 5 for 4 h. Post 4 h, the cells were thoroughly washed with PBS to remove any surface adhered bacteria and a medium containing amikacin (0.2 mg/ml) was added for 2 h to kill any extracellular mycobacteria. After amikacin treatment, the cells thoroughly washed with PBS were taken as 0 h time point and a duplicate set was maintained in antibiotic free medium for next 48 h. Intracellular mycobacteria was enumerated by lysing macrophages with 0.06% SDS in 7H9 Middlebrook medium. Appropriate dilutions were plated onto Middlebrook 7H11 agar plates supplemented with OADC (oleic acid, albumin, dextrose, catalase). Total colony forming units (CFUs) were counted after 21 days of plating.

### Microtomy and Hematoxylin and Eosin (H&E) staining

Microtome sections (5 μm) were obtained from formalin-fixed, paraffin-embedded mouse lung tissue samples using Leica RM2245 microtome. These sections were first deparaffinized and rehydrated. The rehydrated sections were subjected to Hematoxylin staining followed by Eosin staining as per manufacturer instructions. The sections were then dehydrated and mounted with coverslip using permount. Sections were given to consultant pathologists for blinded analyses.

### Cryosection preparation

The excised and fixed lungs were placed in the optimal cutting temperature (OCT) media (Jung, Leica). Cryosections of 10–15 μm were prepared using Leica CM 1510 S or Leica CM 3050 S cryostat with the tissue embedded in OCT being sectioned onto glass slides and then stored at -80°C.

### Immunofluorescence (IF)

Treated/infected macrophages were fixed with 3.6% formaldehyde for 30 min at room temperature. The cells were washed with PBS and blocked in 2% BSA in PBST. After blocking, cells were stained with LRP2 overnight at 4°C. Then they were incubated with DyLight 488-conjugated secondary antibody for 2 h and nuclei were stained with DAPI. The coverslips were mounted on a slide with glycerol. For IF of the cryosections, frozen sections were thawed to room temperature. After blocking with 2% BSA containing saponin, the sections were stained with specific antibodies overnight at 4°C. The sections were then incubated with DyLight 488-conjugated secondary antibody for 2 h and nuclei were stained with DAPI. A coverslip was mounted on the section with glycerol as the medium. Confocal images were taken with Zeiss LSM 710 Meta confocal laser scanning microscope (Carl Zeiss AG, Germany) using a plan-Apochromat 63X/1.4 Oil DIC objective (Carl Zeiss AG, Germany) and images were analyzed using ZEN black software. CTCF (corrected total cell fluorescence) was calculated as (fluorescence observed in an area of a cell–fluorescence of background for the same area) using ImageJ. Cell boundaries were demarcated based on the brightfield image and the fluorescence intensities of different channels were measured. Background fluorescence intensity was measured from a field devoid of cells. For quantitative estimation of the results, at least 100 cells from different fields were analyzed.

### Filipin fluorescence staining for Free Cholesterol

Filipin complex (Sigma-Aldrich, USA) was utilized to assess free cholesterol following protocol from [92]. Briefly, mouse peritoneal macrophages were fixed with 3.6% paraformaldehyde for 1 h at room temperature. After incubation, cells were washed with 1X PBS followed by incubation in 1.5 mg glycine per ml PBS for 10 min at room temperature. Filipin staining was then performed at a final concentration of 0.05 mg/ml in PBS for 2 h at room temperature. Cells were washed thrice with 1X PBS and nuclei were stained with propidium iodide (PI). For Filipin staining of cryosections, frozen sections were thawed to room temperature. After blocking with 2% BSA containing saponin, the sections were stained with Filipin (0.05 mg/ml in PBS) and PE-conjugated F4/80 (macrophage marker) or CD68 AF660 for 2 h at room temperature. The samples were mounted on glycerol. Images were captured in Zeiss LSM 710 confocal laser scanning microscope. CTCF (corrected total cell fluorescence) was calculated as (fluorescence observed in an area of a cell–fluorescence of background for the same area) using ImageJ. Cell boundaries were demarcated based on the brightfield image and the fluorescence intensities of different channels were measured. Background fluorescence intensity was measured from a field devoid of cells. For quantitative estimation of the results, at least 100 cells from different fields were analyzed.

### CellROX Oxidative Stress Reagent staining

CellROX Deep Red Reagent (Thermo Fisher Scientific, USA) was utilized to measure oxidative stress in macrophages as per manufacturer’s instructions. In brief, siRNA transfected mouse peritoneal macrophages were treated with CellROX Deep Red Reagent at a final concentration of 5 mM and incubated for 30 min at 37°C. Cells were then washed with 1X PBS thrice followed by fixation with 3.6% formaldehyde for 15 min. Nuclei were stained with DAPI and images were captured in Zeiss LSM 710 confocal laser scanning microscope. CTCF (corrected total cell fluorescence) was calculated as (fluorescence observed in an area of a cell–fluorescence of background for the same area) using ImageJ. Cell boundaries were demarcated based on the brightfield image and the fluorescence intensities of different channels were measured. Background fluorescence intensity was measured from a field devoid of cells. For quantitative estimation of the results, at least 100 cells from different fields were analyzed.

### Total cholesterol quantification

Total cholesterol levels were quantified using the “Cholesterol Quantification Kit” (Sigma MAK043) per manufacturer’s protocol. Briefly, equal number of cells were seeded for the experiment. Following infection, cells were harvested in 200 microlitre of solution containing chloroform-isopropanol-IGEPAL (7:11:0.1). Organic phase was separated by centrifugation at 13000rpm, followed by air drying at 50°C to remove residual chloroform. Samples were then subjected to Vacuum drying to remove any residual organic solvent. Dried lipids were dissolved in 200 microlitre of cholesterol assay buffer, vortexed and mixed. Appropriate dilutions were made in cholesterol assay buffer for the reaction in 96 well plates. Absorbance was measured at 570nm. Cholesterol standard was prepared, and results were plotted on a standard curve.

### Statistical analysis

Levels of significance for comparison between samples were determined by the Student’s t-test and one-way ANOVA followed by Tukey’s multiple-comparisons. The data in the graphs are expressed as the mean ± SEM for the values from at least 3 or more independent experiments and P values < 0.05 were defined as significant. GraphPad Prism 6.0 software (GraphPad Software, USA) was used for all the statistical analyses.

## Supporting information

S1 Fig Mtb-triggered expression of epigenetic modifiers G9a and SIRT6 in host cells. **(A)** BALB/c peritoneal macrophages were infected with H37Rv for 12 h, and histone modification marks, H3K9me1 and H3K9Ac, were assessed by immunoblotting. **(B)** Transcript level of the *Ehmt2* and *Sirt6* was analyzed by qRT-PCR in lung homogenates of mice infected with H37Rv for 56 days. **(C)** Protein level of G9a and SIRT6 was assessed in BALB/c macrophages infected with H37Rv or *M*. *smegmatis* for 12 h by immunoblotting. **(D)** The protein levels of SIRT6 were assessed in lung homogenates of WT (littermate control), *Sirt6−/+* and *Sirt6−/−* mice by immunoblotting. **(E)** BALB/c mouse peritoneal macrophages were transfected with the indicated siRNAs and infected with H37Rv for 12 h. Whole cell lysates were assessed for the knock down of G9a and SIRT6 by immunoblotting. MTT assay was performed to assess cell viability of BALB/c macrophages transfected with **(F)** NT or *Ehmt2* and *Sirt6* siRNA. The experiments. *, P < 0.05; **P<0.01; ***, P < 0.001 (Student’s t-test for B and F). dium. NT, non-targeting; ns, not significant; WT, wild type.
(TIF)

S2 Fig Mtb-driven free cholesterol accumulation in host cells requires G9a and SIRT6. **(A, B)** BALB/c mouse peritoneal macrophages were infected with H37Rv or *M*. *smegmatis* for 48 h and assessed for cholesterol accumulation by Filipin staining: **(A)** representative image and **(B)** respective quantification. **(C)** Lung cryosections from BALB/c mice infected with H37Rv for 56 days was assessed for cholesterol accumulation by Filipin staining in macrophages stained with F4/80, **(D)** quantification of Filipin staining in F4/80 positive cells in lung cryosections. **(E)** BALB/c mouse peritoneal macrophages transfected with NT or *Ehmt2* or *Sirt6* siRNA were assessed for free cholesterol level upon 48 h infection with tdTomato-expressing H37Rv by immunofluorescence. **(E)** its quantification (n = 200–300). MTT assay was performed to assess cell viability of BALB/c macrophages transfected with **(F)** NT or *Ehmt2* and *Sirt6* siRNA individually, followed by infection with H37Rv for 48h. **(G)** Mouse peritoneal macrophages were transfected with NT, *Ehmt2* or *Sirt6* siRNAs, followed by infection with H37Rv for 48 h. Free cholesterol was assessed using cholesterol estimation kit. **(H, I)** BMDMs from WT (littermate control) and *Sirt6*^−/−^ mice were utilized to assess free cholesterol by Filipin staining upon tdTomato-expressing H37Rv infection for 48 h. **(H)** Representative images and **(I)** its quantification. **(J)** Lung cryosections from uninfected or 56 days H37Rv-infected/ G9a inhibitor (40mg/kg) treated BALB/c mice were assessed for free cholesterol by Filipin staining in macrophages stained with CD68. **(K, L)** Peritoneal macrophages isolated from WT (littermate control) and *Sirt6*^*−/+*^mice were infected with H37Rv and treated with G9a inhibitor (indicated). Total cholesterol was assessed by Filipin staining **(K)** Representative Images and **(L)** respective quantification. **(M)** Lung cryosections of uninfected and infected WT (littermate control) and *Sirt6*^*−/+*^ mice were assessed for free cholesterol levels by Filipin staining in macrophages stained by CD68. The MOI of infection is 1:10 (macrophage: mycobacteria) for all the *in vitro* experiments. All data represents the mean ± SEM from 3 independent experiments. *, P < 0.05; **, P < 0.01; ***, P < 0.001 (Student’s t-test for B, D and F and One-way ANOVA for G, I, J, L, M). Med, Medium. NT, non-targeting; ns, not significant; WT, wild type.
(TIF)

S3 Fig Mtb-driven free cholesterol accumulation in host cells and role of SIRT6. **(A)** Schematic representation of cholesterol biosynthesis pathway. Transcript level of the indicated set of genes was analyzed by qRT-PCR **(B)** in BALB/c mouse peritoneal macrophages infected with H37Rv for 12 h, **(C)** in lung homogenates of BALB/c mice infected with H37Rv for 56 days and **(D)** in human PBMCs infected with H37Rv for 12 h. **(E)** RAW 264.7 macrophages were transfected with vector, or SIRT6 WT construct and transcript levels of ABC transporters was analysed by qRT-PCR. **(F)** BALB/c mouse peritoneal macrophages were transfected with NT or *Sirt6*, followed by 12 h infection with H37Rv. Whole cell lysates were assessed for ABCA1. The MOI of infection is 1:10 (macrophage:mycobacteria) for all the *in vitro* experiments. All data represents the mean ± SEM from 3 independent experiments. *, P < 0.05; **, P < 0.01; ***, P < 0.001 (Student’s t-test for B-E). Med, Medium; PBMC, peripheral blood mononuclear cells.
(TIF)

S4 Fig SREBP2-G9a- and SIRT6 regulate cholesterol metabolism and efflux genes during Mtb infection. **(A)** BALB/c mouse peritoneal macrophages were infected with H37Rv for 12 h and whole cell lysates were assessed for mature SREBP2. **(B-F)** BALB/c mouse peritoneal macrophages were transfected with NT or *Ehmt2* or *Srebf2* or si*Sirt6* siRNA as indicated, followed by 12 h infection with H37Rv. Whole cell lysates were assessed for **(B)** mSREBP2 or **(C)** ABCA1 expression by immunoblotting. **(D-F)** transcript level of the indicated genes was measured by qRT-PCR. **(G)** BALB/c mouse peritoneal macrophages were treated with different concentrations of water-soluble cholesterol for 48h and cholesterol levels were assessed by Filipin staining**. (H)** In vitro CFU was assessed 48h post H37Rv infection under the following condition: BALB/c mouse peritoneal macrophages transiently transfected with siRNAs against *Ehmt2* and *Sirt6* or cholesterol accumulation genes (combination of *Lrp2*, *Aacs*, *Hmgcs1*, *Mvd* and *Dhcr24)* with and without exogenous cholesterol supplementation(50μg) **(I)** MTT assay was performed to assess cell viability of BALB/c macrophages transfected with NT or *Ehmt2* and *Sirt6* or siRNAs against the selected cholesterol accumulation genes (combination of *Lrp2*, *Aacs*, *Hmgcs1*, *Mvd* and *Dhcr24* followed by infection with H37Rv for 48h. The MOI of infection is 1:10 (macrophage: mycobacteria) for all the *in vitro* experiments. All data represents the mean ± SEM from 3 independent experiments. The blots are representative of 3 independent experiments. *, P < 0.05; **, P < 0.01; ***, P < 0.001 ****, P < 0.0001, ns, not significant (One-way ANOVA for D-H) and ns, not significant (Student’s t-test for I) NT, non-targeting; ns, not significant; mSREBP2, mature SREBP.
(TIF)

S5 Fig NRF2 and its target genes are expressed during Mtb infection. **(A)** BALB/c mouse peritoneal macrophages were infected with H37Rv for 48 h and the expression of NRF2 target genes was assessed by qRT-PCR. **(B)** BALB/c mouse peritoneal macrophages were infected with H37Rv for the indicated time points and whole cell lysates were assessed for the expression of NRF2 and its target genes. **(C)** Immunoblotting to validate NRF2 knockdown in murine macrophages transfected with *Nfe2l2* siRNA. **(D-F)** BALB/c mouse peritoneal macrophages were transfected with NT or **(D)** *Nfe2l2* siRNA or **(E)** *Ehmt2* and *Sirt6* siRNA or Chol accum genes siRNA (combination of *Lrp2*, *Aacs*, *Hmgcs1*, *Mvd* and *Dhcr24* siRNAs) or **(F)** siRNAs against the selected cholesterol accumulation genes (combination of *Lrp2*, *Aacs*, *Hmgcs1*, *Mvd* and *Dhcr24* siRNAs) or NT and assessed for the indicated transcript by qRT-PCR. **(G)** MTT assay was performed to assess cell viability of BALB/c macrophages transfected with NT or siRNA against selected cholesterol accumulation genes. **(H-I)** BALB/c mouse peritoneal macrophages were infected with H37Rv for 48 h and stained with CellROX to assess for oxidative stress was performed. Representative images and **(H)** its quantification **(I). (J)** BALB/c mouse peritoneal macrophages were transfected with siRNAs against the selected cholesterol accumulation genes (combination of *Lrp2*, *Aacs*, *Hmgcs1*, *Mvd* and *Dhcr24* siRNAs) or NT and followed by H37Rv infection for 48 h and cholesterol accumulation was confirmed by Filipin staining;. The MOI of infection is 1:10 (macrophage:mycobacteria) for all the *in vitro* experiments. All data represents the mean ± SEM from 3 independent experiments; *, P < 0.05; **, P < 0.01; ***, P < 0.001 (Student’s t- test for A, G, I and One-Way ANOVA for D-F, J) and the blots are representative of 3 independent experiments. Med, Medium; NT, non-targeting; ns, not significant; chol. accum. genes, cholesterol accumulation genes.
(TIF)

S6 Fig Histological evaluation of Mtb infected murine lungs. Lungs of BALB/c mice were subjected to histological evaluation for TB pathology by scoring for % of granulomatous area for **(A)** G9a inhibitor (40mg/kg) treated and untreated BALB/c mice after 56 days of total H37Rv infection and therapeutic treatment and **(B)** WT (littermate control) and *Sirt6*^*−/+*^ mice. **, p<0.01, Student’s t-test.
(TIF)

S1 File List of all primers used in manuscript has been provided. (PDF)

S2 File RAW data for all the Immunoblotting experiments have been provided as a supplemental document. (PDF)

## References

[1] Cole J, Morris P, Dickman MJ, Dockrell DH. The therapeutic potential of epigenetic manipulation during infectious diseases. Pharmacology and Therapeutics. 2016;167: 85–99. doi: 10.1016/j.pharmthera.2016.07.013 27519803 PMC5109899

[2] Esterhuyse MM, Linhart HG, Kaufmann SHE. Can the battle against tuberculosis gain from epigenetic research? Trends in Microbiology. 2012;20: 220–226. doi: 10.1016/j.tim.2012.03.002 22464289

[3] Kathirvel M, Mahadevan S. The role of epigenetics in tuberculosis infection. Epigenomics. Future Medicine Ltd.; 2016. pp. 537–549. doi: 10.2217/epi.16.1 27035266

[4] Ghorpade DS, Holla S, Sinha AY, Alagesan SK, Balaji KN. Nitric oxide and KLF4 protein epigenetically modify class II transactivator to repress major histocompatibility complex II expression during Mycobacterium bovis bacillus Calmette-Guérin infection. Journal of Biological Chemistry. 2013;288: 20592–20606. doi: 10.1074/jbc.M113.472183 23733190 PMC3711323

[5] Singh V, Prakhar P, Rajmani RS, Mahadik K, Borbora SM, Balaji KN. Histone methyltransferase SET8 epigenetically reprograms host immune responses to assist mycobacterial survival. Journal of Infectious Diseases. 2017;216: 477–488. doi: 10.1093/infdis/jix322 28931237

[6] Yaseen I, Kaur P, Nandicoori VK, Khosla S. Mycobacteria modulate host epigenetic machinery by Rv1988 methylation of a non-tail arginine of histone H3. Nature Communications. 2015;6: 1–13. doi: 10.1038/ncomms9922 26568365

[7] Russell DG, Cardona PJ, Kim MJ, Allain S, Altare F. Foamy macrophages and the progression of the human tuberculosis granuloma. Nature Immunology. NIH Public Access; 2009. pp. 943–948. doi: 10.1038/ni.1781 19692995 PMC2759071

[8] Holla S, Prakhar P, Singh V, Karnam A, Mukherjee T, Mahadik K, et al. MUSASHI-Mediated Expression of JMJD3, a H3K27me3 Demethylase, Is Involved in Foamy Macrophage Generation during Mycobacterial Infection. Fortune SM, editor. PLOS Pathogens. 2016;12: e1005814. doi: 10.1371/journal.ppat.1005814 27532872 PMC4988650

[9] Knight M, Braverman J, Asfaha K, Gronert K, Stanley S. Lipid droplet formation in Mycobacterium tuberculosis infected macrophages requires IFN-γ/HIF-1α signaling and supports host defense. PLoS Pathogens. 2018. doi: 10.1371/journal.ppat.1006874 29370315 PMC5800697

[10] Chandra P, He L, Zimmerman M, Yang G, Köster S, Ouimet M, et al. Inhibition of fatty acid oxidation promotes macrophage control of mycobacterium tuberculosis. mBio. 2020;11: 1–15. doi: 10.1128/mBio.01139-20 32636249 PMC7343992

[11] Daniel J, Maamar H, Deb C, Sirakova TD, Kolattukudy PE. Mycobacterium tuberculosis uses host triacylglycerol to accumulate lipid droplets and acquires a dormancy-like phenotype in lipid-loaded macrophages. PLoS Pathogens. 2011;7. doi: 10.1371/journal.ppat.1002093 21731490 PMC3121879

[12] D’Avila H, Melo RCN, Parreira GG, Werneck-Barroso E, Castro-Faria-Neto HC, Bozza PT. Mycobacterium bovis Bacillus Calmette-Guérin Induces TLR2-Mediated Formation of Lipid Bodies: Intracellular Domains for Eicosanoid Synthesis In Vivo. The Journal of Immunology. 2006;176: 3087–3097. doi: 10.4049/jimmunol.176.5.3087 16493068

[13] Dodd CE, Pyle CJ, Glowinski R, Rajaram MVS, Schlesinger LS. CD36-Mediated Uptake of Surfactant Lipids by Human Macrophages Promotes Intracellular Growth of Mycobacterium tuberculosis. The Journal of Immunology. 2016;197: 4727–4735. doi: 10.4049/jimmunol.1600856 27913648 PMC5137803

[14] Kim MJ, Wainwright HC, Locketz M, Bekker LG, Walther GB, Dittrich C, et al. Caseation of human tuberculosis granulomas correlates with elevated host lipid metabolism. EMBO Molecular Medicine. 2010;2: 258–274. doi: 10.1002/emmm.201000079 20597103 PMC2913288

[15] Peyron P, Vaubourgeix J, Poquet Y, Levillain F, Botanch C, Bardou F, et al. Foamy macrophages from tuberculous patients’ granulomas constitute a nutrient-rich reservoir for M. tuberculosis persistence. PLoS Pathogens. 2008;4. doi: 10.1371/journal.ppat.1000204 19002241 PMC2575403

[16] Singh V, Jamwal S, Jain R, Verma P, Gokhale R, Rao KVS. Mycobacterium tuberculosis-driven targeted recalibration of macrophage lipid homeostasis promotes the foamy phenotype. Cell Host and Microbe. 2012;12: 669–681. doi: 10.1016/j.chom.2012.09.012 23159056

[17] Brzostek A, Pawelczyk J, Rumijowska-Galewicz A, Dziadek B, Dziadek J. Mycobacterium tuberculosis is able to accumulate and utilize cholesterol. Journal of Bacteriology. 2009. doi: 10.1128/JB.00488-09 19717592 PMC2795286

[18] Ouimet M, Koster S, Sakowski E, Ramkhelawon B, Van Solingen C, Oldebeken S, et al. Mycobacterium tuberculosis induces the MIR-33 locus to reprogram autophagy and host lipid metabolism. Nature Immunology. 2016;17: 677–686. doi: 10.1038/ni.3434 27089382 PMC4873392

[19] Kim YS, Lee H-M, Kim JK, Yang C-S, Kim TS, Jung M, et al. PPAR-$α$ Activation Mediates Innate Host Defense through Induction of TFEB and Lipid Catabolism. The Journal of Immunology. 2017;198: 3283–3295. doi: 10.4049/jimmunol.1601920 28275133

[20] Gatfield J, Pieters J. Essential role for cholesterol in entry of mycobacteria into macrophages. Science. 2000;288: 1647–1650. doi: 10.1126/science.288.5471.1647 10834844

[21] Lee W, VanderVen BC, Fahey RJ, Russell DG. Intracellular Mycobacterium tuberculosis exploits host-derived fatty acids to limit metabolic stress. Journal of Biological Chemistry. 2013;288: 6788–6800. doi: 10.1074/jbc.M112.445056 23306194 PMC3591590

[22] Meaney S. Epigenetic regulation of cholesterol homeostasis. Frontiers in Genetics. 2014;5: 311. doi: 10.3389/fgene.2014.00311 25309573 PMC4174035

[23] Rayner KJ, Sheedy FJ, Esau CC, Hussain FN, Temel RE, Parathath S, et al. Antagonism of miR-33 in mice promotes reverse cholesterol transport and regression of atherosclerosis. Journal of Clinical Investigation. 2011;121: 2921–2931. doi: 10.1172/JCI57275 21646721 PMC3223840

[24] K, Sowers ML, Cherryhomes EI, Singh VK, Mishra A, Restrepo BI, et al. Sirtuin-dependent metabolic and epigenetic regulation of macrophages during tuberculosis. Front Immunol. 2023 Mar 13;14:1121495. doi: 10.3389/fimmu.2023.1121495 36993975 PMC10040548

[25] Cardoso F, Castro F, Moreira-Teixeira L, Sousa J, Torrado E, Silvestre R, et al. Myeloid Sirtuin 2 Expression Does Not Impact Long-Term Mycobacterium tuberculosis Control. Cardona P-J, editor. PLOS ONE. 2015;10: e0131904. doi: 10.1371/journal.pone.0131904 26135889 PMC4489762

[26] Cheng CY, Gutierrez NM, Marzuki MB, Lu X, Foreman TW, Paleja B, et al. Host sirtuin 1 regulates mycobacterial immunopathogenesis and represents a therapeutic target against tuberculosis. Science Immunology. 2017;2. doi: 10.1126/sciimmunol.aaj1789 28707004 PMC5505666

[27] Ghosh HS, Reizis B, Robbins PD. SIRT1 associates with eIF2-alpha and regulates the cellular stress response. Scientific Reports. 2011. doi: 10.1038/srep00150 22355666 PMC3252071

[28] Hayakawa T, Iwai M, Aoki S, Takimoto K, Maruyama M, Maruyama W, et al. SIRT1 suppresses the senescence-associated secretory phenotype through epigenetic gene regulation. PLoS ONE. 2015;10. doi: 10.1371/journal.pone.0116480 25635860 PMC4312089

[29] Hou X, Xu S, Maitland-Toolan KA, Sato K, Jiang B, Ido Y, et al. SIRT1 regulates hepatocyte lipid metabolism through activating AMP-activated protein kinase. Journal of Biological Chemistry. 2008;283: 20015–20026. doi: 10.1074/jbc.M802187200 18482975 PMC2459285

[30] Liu TF, McCall CE. Deacetylation by SIRT1 Reprograms Inflammation and Cancer. Genes and Cancer. 2013;4: 135–147. doi: 10.1177/1947601913476948 24020005 PMC3764465

[31] Yuan H, Wang Z, Li L, Zhang H, Modi H, Horne D, et al. Activation of stress response gene SIRT1 by BCR-ABL promotes leukemogenesis. Blood. 2012;119: 1904–1914. doi: 10.1182/blood-2011-06-361691 22207735 PMC3293644

[32] Shi L, Jiang Q, Bushkin Y, Subbian S, Tyagi S. Biphasic dynamics of macrophage immunometabolism during Mycobacterium tuberculosis infection. mBio. 2019;10. doi: 10.1128/mBio.02550-18 30914513 PMC6437057

[33] Dang W. The controversial world of sirtuins. Drug Discovery Today: Technologies. Elsevier Ltd; 2014. p. e9. doi: 10.1016/j.ddtec.2012.08.003 25027380 PMC4101544

[34] Kanfi Y, Naiman S, Amir G, Peshti V, Zinman G, Nahum L, et al. The sirtuin SIRT6 regulates lifespan in male mice. Nature. 2012;483: 218–221. doi: 10.1038/nature10815 22367546

[35] Sebastián C, Zwaans BMM, Silberman DM, Gymrek M, Goren A, Zhong L, et al. The histone deacetylase SIRT6 Is a tumor suppressor that controls cancer metabolism. Cell. 2012;151: 1185–1199. doi: 10.1016/j.cell.2012.10.047 23217706 PMC3526953

[36] Wu X, Cao N, Fenech M, Wang X. Role of Sirtuins in Maintenance of Genomic Stability: Relevance to Cancer and Healthy Aging. DNA and Cell Biology. Mary Ann Liebert Inc.; 2016. pp. 542–575. doi: 10.1089/dna.2016.3280 27380140

[37] Elhanati S, Kanfi Y, Varvak A, Roichman A, Carmel-Gross I, Barth S, et al. Multiple regulatory layers of SREBP1/2 by SIRT6. Cell Reports. 2013;4: 905–912. doi: 10.1016/j.celrep.2013.08.006 24012758

[38] Gazzar M El Yoza BK, Chen X Garcia BA, Young NL McCall CE. Chromatin-Specific Remodeling by HMGB1 and Linker Histone H1 Silences Proinflammatory Genes during Endotoxin Tolerance. Molecular and Cellular Biology. 2009;29: 1959–1971. doi: 10.1128/MCB.01862-08 19158276 PMC2655606

[39] El Gazzar M, Yoza BK, Chen X, Hu J, Hawkins GA, McCall CE. G9a and HP1 couple histone and DNA methylation to TNF$α$ transcription silencing during endotoxin tolerance. Journal of Biological Chemistry. 2008;283: 32198–32208. doi: 10.1074/jbc.M803446200 18809684 PMC2583293

[40] Imai K, Togami H, Okamoto T. Involvement of histone H3 lysine 9 (H3K9) methyltransferase G9a in the maintenance of HIV-1 latency and its reactivation by BIX01294. Journal of Biological Chemistry. 2010;285: 16538–16545. doi: 10.1074/jbc.M110.103531 20335163 PMC2878073

[41] Merkling SH, Bronkhorst AW, Kramer JM, Overheul GJ, Schenck A, Van Rij RP. The Epigenetic Regulator G9a Mediates Tolerance to RNA Virus Infection in Drosophila. PLoS Pathogens. 2015;11. doi: 10.1371/journal.ppat.1004692 25880195 PMC4399909

[42] Scheer S, Zaph C. The lysine methyltransferase G9a in immune cell differentiation and function. Frontiers in Immunology. Frontiers Research Foundation; 2017. p. 429. doi: 10.3389/fimmu.2017.00429 28443098 PMC5387087

[43] Tachibana M, Sugimoto K, Nozaki M, Ueda J, Ohta T, Ohki M, et al. G9a histone methyltransferase plays a dominant role in euchromatic histone H3 lysine 9 methylation and is essential for early embryogenesis. Genes and Development. 2002;16: 1779–1791. doi: 10.1101/gad.989402 12130538 PMC186403

[44] Mukherjee T, Balaji KN. The WNT framework in shaping immune cell responses during bacterial infections. Frontiers in Immunology. 2019. doi: 10.3389/fimmu.2019.01985 31497020 PMC6712069

[45] Villaseñor T, Madrid-Paulino E, Maldonado-Bravo R, Urbán-Aragón A, Pérez-Martínez L, Pedraza-Alva G. Activation of the Wnt pathway by Mycobacterium tuberculosis: A Wnt-Wnt Situation. Frontiers in Immunology. 2017. doi: 10.3389/fimmu.2017.00050 28203237 PMC5285348

[46] Mostoslavsky R, Chua KF, Lombard DB, Pang WW, Fischer MR, Gellon L, et al. Genomic instability and aging-like phenotype in the absence of mammalian SIRT6. Cell. 2006;124: 315–329. doi: 10.1016/j.cell.2005.11.044 16439206

[47] Espenshade PJ. SREBPs: Sterol-regulated transcription factors. Journal of Cell Science. 2006;119: 973–976. doi: 10.1242/jcs.02866 16525117

[48] DeVries-Seimon T, Li Y, Pin MY, Stone E, Wang Y, Davis RJ, et al. Cholesterol-induced macrophage apoptosis requires ER stress pathways and engagement of the type A scavenger receptor. Journal of Cell Biology. 2005;171: 61–73. doi: 10.1083/jcb.200502078 16203857 PMC2171235

[49] Jin X, Xu Z, Cao J, Yan R, Xu R, Ran R, et al. HO-1/EBP interaction alleviates cholesterol-induced hypoxia through the activation of the AKT and Nrf2/mTOR pathways and inhibition of carbohydrate metabolism in cardiomyocytes. International Journal of Molecular Medicine. 2017;39: 1409–1420. doi: 10.3892/ijmm.2017.2979 28487965 PMC5428940

[50] Kaminsky-Kolesnikov Y, Rauchbach E, Abu-Halaka D, Hahn M, García-Ruiz C, Fernandez-Checa JC, et al. Cholesterol Induces Nrf-2-and HIF-1 α-Dependent Hepatocyte Proliferation and Liver Regeneration to Ameliorate Bile Acid Toxicity in Mouse Models of NASH and Fibrosis. Oxidative Medicine and Cellular Longevity. 2020. doi: 10.1155/2020/5393761 32566088 PMC7271232

[51] Thimmulappa RK, Lee H, Rangasamy T, Reddy SP, Yamamoto M, Kensler TW, et al. Nrf2 is a critical regulator of the innate immune response and survival during experimental sepsis. Journal of Clinical Investigation. 2006;116: 984. doi: 10.1172/JCI25790 16585964 PMC1421348

[52] Freigang S, Ampenberger F, Spohn G, Heer S, Shamshiev AT, Kisielow J, et al. Nrf2 is essential for cholesterol crystal-induced inflammasome activation and exacerbation of atherosclerosis. European Journal of Immunology. 2011. doi: 10.1002/eji.201041316 21484785

[53] Zhao X, Khan N, Gan H, Tzelepis F, Nishimura T, Park SY, et al. Bcl-xL mediates RIPK3-dependent necrosis in M. tuberculosis-infected macrophages. Mucosal Immunology 2017 10:6. 2017;10: 1553–1568. doi: 10.1038/mi.2017.12 28401933 PMC5638669

[54] Borbora SM, Rajmani RS, Balaji KN. PRMT5 epigenetically regulates the E3 ubiquitin ligase ITCH to influence lipid accumulation during mycobacterial infection. PLoS Pathogens. 2022;18: e1010095. doi: 10.1371/journal.ppat.1010095 35658060 PMC9200362

[55] Boro M, Singh V, Balaji KN. Mycobacterium tuberculosis-triggered Hippo pathway orchestrates CXCL1/2 expression to modulate host immune responses. Scientific Reports 2016 6:1. 2016;6: 1–14. doi: 10.1038/srep37695 27883091 PMC5121601

[56] Holla S, Stephen-Victor E, Prakhar P, Sharma M, Saha C, Udupa V, et al. Mycobacteria-responsive sonic hedgehog signaling mediates programmed death-ligand 1- and prostaglandin E2-induced regulatory T cell expansion. Scientific Reports. 2016;6. doi: 10.1038/SREP24193 27080341 PMC4832185

[57] Bell KFS, Al-Mubarak B, Martel MA, McKay S, Wheelan N, Hasel P, et al. Neuronal development is promoted by weakened intrinsic antioxidant defences due to epigenetic repression of Nrf2. Nature Communications. 2015;6: 1–15. doi: 10.1038/ncomms8066 25967870 PMC4441249

[58] Rada P, Rojo AI, Offergeld A, Feng GJ, Velasco-Martín JP, González-Sancho JM, et al. WNT-3A regulates an Axin1/NRF2 complex that regulates antioxidant metabolism in hepatocytes. Antioxidants and Redox Signaling. 2015;22: 555–571. doi: 10.1089/ars.2014.6040 25336178 PMC4333636

[59] Brandenburg J, Marwitz S, Tazoll SC, Waldow F, Kalsdorf B, Vierbuchen T, et al. WNT6/ACC2-induced storage of triacylglycerols in macrophages is exploited by Mycobacterium tuberculosis. The Journal of Clinical Investigation. 2021;131. doi: 10.1172/JCI141833 34255743 PMC8363280

[60] Scott CC, Vossio S, Vacca F, Snijder B, Larios J, Schaad O, et al. Wnt directs the endosomal flux of LDL -derived cholesterol and lipid droplet homeostasis. EMBO reports. 2015;16: 741–752. doi: 10.15252/embr.201540081 25851648 PMC4467858

[61] Mohn WW, Van Der Geize R, Stewart GR, Okamoto S, Liu J, Dijkhuizen L, et al. The actinobacterial mce4 locus encodes a steroid transporter. Journal of Biological Chemistry. 2008;283: 35368–35374. doi: 10.1074/jbc.M805496200 18955493 PMC5218832

[62] Nazarova E V, Montague CR, La T, Wilburn KM, Sukumar N, Lee W, et al. Rv3723/LucA coordinates fatty acid and cholesterol uptake in Mycobacterium tuberculosis. eLife. 2017;6. doi: 10.7554/eLife.26969 28708968 PMC5487216

[63] Pandey AK, Sassetti CM. Mycobacterial persistence requires the utilization of host cholesterol. Proceedings of the National Academy of Sciences of the United States of America. 2008;105: 4376–4380. doi: 10.1073/pnas.0711159105 18334639 PMC2393810

[64] de Chastellier C, Thilo L. Cholesterol depletion in Mycobacterium avium-infected macrophages overcomes the block in phagosome maturation and leads to the reversible sequestration of viable mycobacteria in phagolysosome-derived autophagic vacuoles. Cellular Microbiology. 2006;8: 242–256. doi: 10.1111/j.1462-5822.2005.00617.x 16441435

[65] Lin HH, Wu CY, Wang CH, Fu H, Lönnroth K, Chang YC, et al. Association of obesity, diabetes, and risk of tuberculosis: Two population-based cohorts. Clinical Infectious Diseases. 2018;66: 699–705. doi: 10.1093/cid/cix852 29029077 PMC5850624

[66] Martens GW, Arikan MC, Lee J, Ren F, Vallerskog T, Kornfeld H. Hypercholesterolemia impairs immunity to tuberculosis. Infection and Immunity. 2008;76: 3464–3472. doi: 10.1128/IAI.00037-08 18505807 PMC2493195

[67] Martens GW, Vallerskog T, Kornfeld H. Hypercholesterolemic LDL receptor-deficient mice mount a neutrophilic response to tuberculosis despite the timely expression of protective immunity. Journal of Leukocyte Biology. 2012;91: 849–857. doi: 10.1189/jlb.0311164 22227965 PMC3360472

[68] Shimabukuro-Vornhagen A, Zoghi S, Liebig TM, Wennhold K, Chemitz J, Draube A, et al. Inhibition of Protein Geranylgeranylation Specifically Interferes with CD40-Dependent B Cell Activation, Resulting in a Reduced Capacity To Induce T Cell Immunity. The Journal of Immunology. 2014;193: 5294–5305. doi: 10.4049/jimmunol.1203436 25311809

[69] Yang W, Bai Y, Xiong Y, Zhang J, Chen S, Zheng X, et al. Potentiating the antitumour response of CD8+ T cells by modulating cholesterol metabolism. Nature. 2016;531: 651–655. doi: 10.1038/nature17412 26982734 PMC4851431

[70] Ito A, Hong C, Oka K, Salazar J V, Diehl C, Witztum JL, et al. Cholesterol Accumulation in CD11c+ Immune Cells Is a Causal and Targetable Factor in Autoimmune Disease. Immunity. 2016;45: 1311–1326. doi: 10.1016/j.immuni.2016.11.008 28002731 PMC5181791

[71] Kim KD, Lim HY, Lee HG, Yoon DY, Choe YK, Choi I, et al. Apolipoprotein A-I induces IL-10 and PGE2 production in human monocytes and inhibits dendritic cell differentiation and maturation. Biochemical and Biophysical Research Communications. 2005;338: 1126–1136. doi: 10.1016/j.bbrc.2005.10.065 16259956

[72] Swirski FK, Libby P, Aikawa E, Alcaide P, Luscinskas FW, Weissleder R, et al. Ly-6Chi monocytes dominate hypercholesterolemia-associated monocytosis and give rise to macrophages in atheromata. Journal of Clinical Investigation. 2007;117: 195–205. doi: 10.1172/JCI29950 17200719 PMC1716211

[73] Yvan-Charvet L, Pagler TA, Seimon TA, Thorp E, Welch CL, Witztum JL, et al. ABCA1 and ABCG1 protect against oxidative stress-induced macrophage apoptosis during efferocytosis. Circulation Research. 2010;106: 1861–1869. doi: 10.1161/CIRCRESAHA.110.217281 20431058 PMC2995809

[74] Zhu X, Lee JY, Timmins JM, Brown JM, Boudyguina E, Mulya A, et al. Increased cellular free cholesterol in macrophage-specific Abca1 knock-out mice enhances pro-inflammatory response of macrophages. Journal of Biological Chemistry. 2008;283: 22930–22941. doi: 10.1074/jbc.M801408200 18552351 PMC2516976

[75] Zhu X, Owen JS, Wilson MD, Li H, Griffiths GL, Thomas MJ, et al. Macrophage ABCA1 reduces MyD88-dependent toll-like receptor trafficking to lipid rafts by reduction of lipid raft cholesterol. Journal of Lipid Research. 2010;51: 3196–3206. doi: 10.1194/jlr.M006486 20650929 PMC2952560

[76] Huang FC. The critical role of membrane cholesterol in Salmonella-induced autophagy in intestinal epithelial cells. International Journal of Molecular Sciences. 2014;15: 12558–12572. doi: 10.3390/ijms150712558 25029544 PMC4139860

[77] Abrams ME, Johnson KA, Perelman SS, shu Zhang L, Endapally S, Mar KB, et al. Oxysterols provide innate immunity to bacterial infection by mobilizing cell surface accessible cholesterol. Nature Microbiology. 2020;5: 929–942. doi: 10.1038/s41564-020-0701-5 32284563 PMC7442315

[78] Ahsan F, Maertzdorf J, Guhlich-Bornhof U, Kaufmann SHE, Moura-Alves P. IL-36/LXR axis modulates cholesterol metabolism and immune defense to Mycobacterium tuberculosis. Scientific Reports. 2018;8: 1520. doi: 10.1038/s41598-018-19476-x 29367626 PMC5784124

[79] Korf H, Vander Beken S, Romano M, Steffensen KR, Stijlemans B, Gustafsson JÅ, et al. Liver X receptors contribute to the protective immune response against Mycobacterium tuberculosis in mice. Journal of Clinical Investigation. 2009;119: 1626–1637. doi: 10.1172/JCI35288 19436111 PMC2689129

[80] He J, Zhang G, Pang Q, Yu C, Xiong J, Zhu J, et al. SIRT6 reduces macrophage foam cell formation by inducing autophagy and cholesterol efflux under ox-LDL condition. The FEBS Journal. 2017;284: 1324–1337. doi: 10.1111/febs.14055 28296196

[81] Wang L, Xu S, Lee JE, Baldridge A, Grullon S, Peng W, et al. Histone H3K9 methyltransferase G9a represses PPAR$γ$ expression and adipogenesis. EMBO Journal. 2013;32: 45–59. doi: 10.1038/emboj.2012.306 23178591 PMC3545301

[82] Rajaram MVS, Brooks MN, Morris JD, Torrelles JB, Azad AK, Schlesinger LS. Mycobacterium tuberculosis activates human macrophage peroxisome proliferator-activated receptor gamma linking mannose receptor recognition to regulation of immune responses. Journal of immunology (Baltimore, Md: 1950). 2010;185: 929–942. doi: 10.4049/jimmunol.1000866 20554962 PMC3014549

[83] Jin X, Xu Z, Fan R, Wang C, Ji W, Ma Y, et al. HO-1 alleviates cholesterol-induced oxidative stress through activation of Nrf2/ERK and inhibition of PI3K/AKT pathways in endothelial cells. Molecular Medicine Reports. 2017;16: 3519–3527. doi: 10.3892/mmr.2017.6962 28713890

[84] Parihar SP, Guler R, Khutlang R, Lang DM, Hurdayal R, Mhlanga MM, et al. Statin therapy reduces the mycobacterium tuberculosis burden in human macrophages and in mice by enhancing autophagy and phagosome maturation. Journal of Infectious Diseases. 2014;209: 754–763. doi: 10.1093/infdis/jit550 24133190

[85] Bhaskar A, Kumar S, Khan MZ, Singh A, Dwivedi VP, Nandicoori VK. Host sirtuin 2 as an immunotherapeutic target against tuberculosis. eLife. 2020. doi: 10.7554/eLife.55415 32697192 PMC7398663

[86] Hamers AAJ, Argmann C, Moerland PD, Koenis DS, Marinković G, Sokolović M, et al. Nur77-deficiency in bone marrow-derived macrophages modulates inflammatory responses, extracellular matrix homeostasis, phagocytosis and tolerance. BMC Genomics. 2016;17: 162. doi: 10.1186/s12864-016-2469-9 26932821 PMC4774191

[87] Zhang X, Edwards JP, Mosser DM. The expression of exogenous genes in macrophages: Obstacles and opportunities. Methods in Molecular Biology. 2009;531: 123–143. doi: 10.1007/978-1-59745-396-7_9 19347315 PMC2821576

[88] Malmquist NA, Moss TA, Mecheri S, Scherf A, Fuchter MJ. Small-molecule histone methyltransferase inhibitors display rapid antimalarial activity against all blood stage forms in Plasmodium falciparum. Proceedings of the National Academy of Sciences of the United States of America. 2012;109: 16708–16713. doi: 10.1073/pnas.1205414109 23011794 PMC3478629

[89] Singh R, Singh M, Arora G, Kumar S, Tiwari P, Kidwai S. Polyphosphate deficiency in Mycobacterium tuberculosis is associated with enhanced drug susceptibility and impaired growth in guinea pigs. Journal of Bacteriology. 2013;195: 2839–2851. doi: 10.1128/JB.00038-13 23585537 PMC3697247

[90] Truax AD, Greer SF. ChIP and Re-ChIP assays: investigating interactions between regulatory proteins, histone modifications, and the DNA sequences to which they bind. Methods in molecular biology (Clifton, NJ). 2012;809: 175–188. doi: 10.1007/978-1-61779-376-9_12 22113276

[91] De Medeiros RB. Sequential chromatin immunoprecipitation assay and analysis. Methods in molecular biology (Clifton, NJ). 2011;791: 225–237. doi: 10.1007/978-1-61779-316-5_17 21913083

[92] Leventhal AR, Chen W, Tall AR, Tabas I. Acid sphingomyelinase-deficient macrophages have defective cholesterol trafficking and efflux. The Journal of biological chemistry. 2001;276: 44976–44983. doi: 10.1074/jbc.M106455200 11579092

