# Supplementary figures and images for: G9a and Sirtuin6 epigenetically modulate host cholesterol accumulation to facilitate mycobacterial survival
Source: PLoS Pathog. 2023 Oct 23;19(10):e1011731. doi: 10.1371/journal.ppat.1011731 (PMC10621959; doi:10.1371/journal.ppat.1011731)

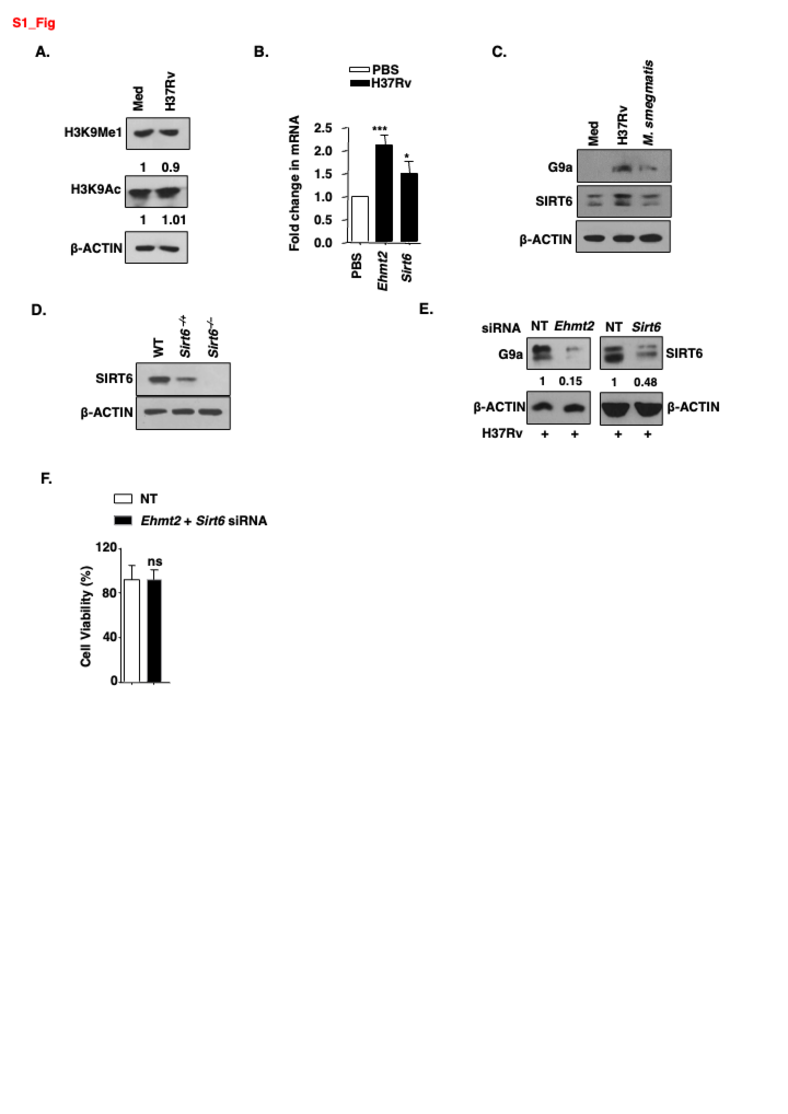

Supplement: S1 Fig — (A) BALB/c peritoneal macrophages were infected with H37Rv for 12 h, and histone modification marks, H3K9me1 and H3K9Ac, were assessed by immunoblotting. (B) Transcript level of the Ehmt2 and Sirt6 was analyzed by qRT-PCR in lung homogenates of mice infected with H37Rv for 56 days. (C) Protein level of G9a and SIRT6 was assessed in BALB/c macrophages infected with H37Rv or M. smegmatis for 12 h by immunoblotting. (D) The protein levels of SIRT6 were assessed in lung homogenates of WT (littermate control), Sirt6−/+ and Sirt6−/− mice by immunoblotting. (E) BALB/c mouse peritoneal macrophages were transfected with the indicated siRNAs and infected with H37Rv for 12 h. Whole cell lysates were assessed for the knock down of G9a and SIRT6 by immunoblotting. MTT assay was performed to assess cell viability of BALB/c macrophages transfected with (F) NT or Ehmt2 and Sirt6 siRNA. The experiments. *, P < 0.05; **P<0.01; ***, P < 0.001 (Student’s t-test for B and F). dium. NT, non-targeting; ns, not significant; WT, wild type. (TIF) [file ppat.1011731.s001.tif]

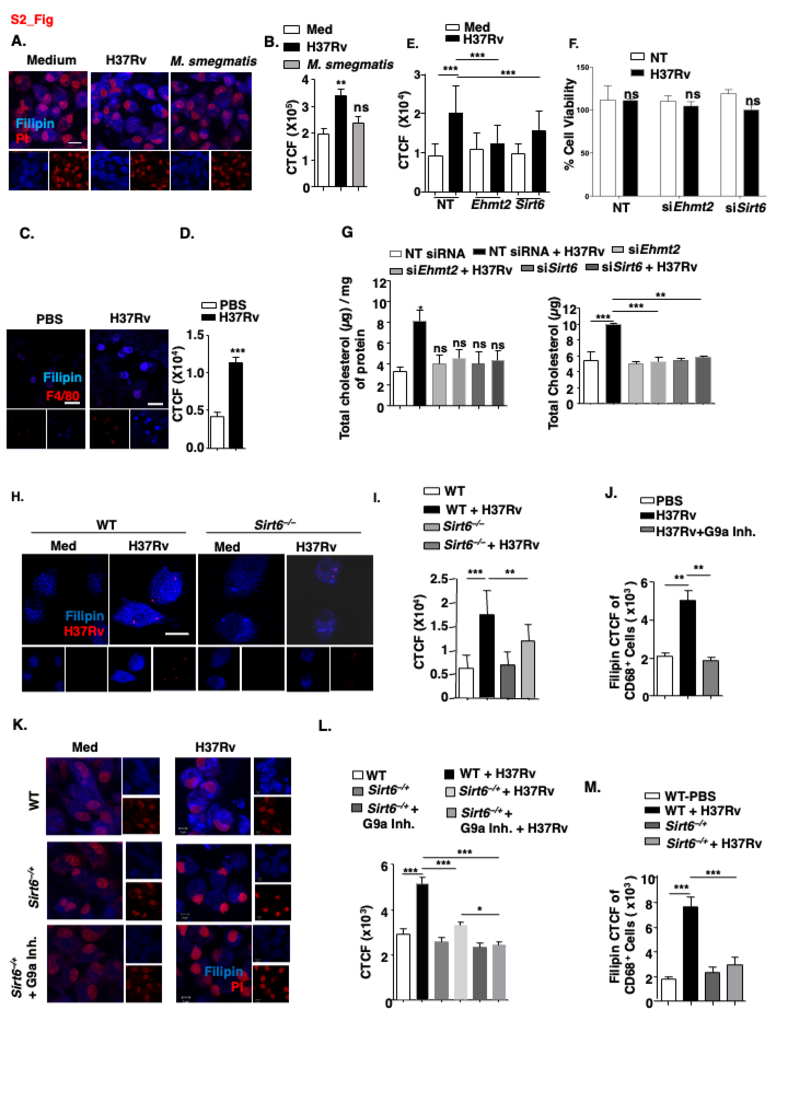

Supplement: S2 Fig — (A, B) BALB/c mouse peritoneal macrophages were infected with H37Rv or M. smegmatis for 48 h and assessed for cholesterol accumulation by Filipin staining: (A) representative image and (B) respective quantification. (C) Lung cryosections from BALB/c mice infected with H37Rv for 56 days was assessed for cholesterol accumulation by Filipin staining in macrophages stained with F4/80, (D) quantification of Filipin staining in F4/80 positive cells in lung cryosections. (E) BALB/c mouse peritoneal macrophages transfected with NT or Ehmt2 or Sirt6 siRNA were assessed for free cholesterol level upon 48 h infection with tdTomato-expressing H37Rv by immunofluorescence. (E) its quantification (n = 200–300). MTT assay was performed to assess cell viability of BALB/c macrophages transfected with (F) NT or Ehmt2 and Sirt6 siRNA individually, followed by infection with H37Rv for 48h. (G) Mouse peritoneal macrophages were transfected with NT, Ehmt2 or Sirt6 siRNAs, followed by infection with H37Rv for 48 h. Free cholesterol was assessed using cholesterol estimation kit. (H, I) BMDMs from WT (littermate control) and Sirt6−/− mice were utilized to assess free cholesterol by Filipin staining upon tdTomato-expressing H37Rv infection for 48 h. (H) Representative images and (I) its quantification. (J) Lung cryosections from uninfected or 56 days H37Rv-infected/ G9a inhibitor (40mg/kg) treated BALB/c mice were assessed for free cholesterol by Filipin staining in macrophages stained with CD68. (K, L) Peritoneal macrophages isolated from WT (littermate control) and Sirt6−/+mice were infected with H37Rv and treated with G9a inhibitor (indicated). Total cholesterol was assessed by Filipin staining (K) Representative Images and (L) respective quantification. (M) Lung cryosections of uninfected and infected WT (littermate control) and Sirt6−/+ mice were assessed for free cholesterol levels by Filipin staining in macrophages stained by CD68. The MOI of infection is 1:10 (macrophage: mycobacteria [file ppat.1011731.s002.tif]

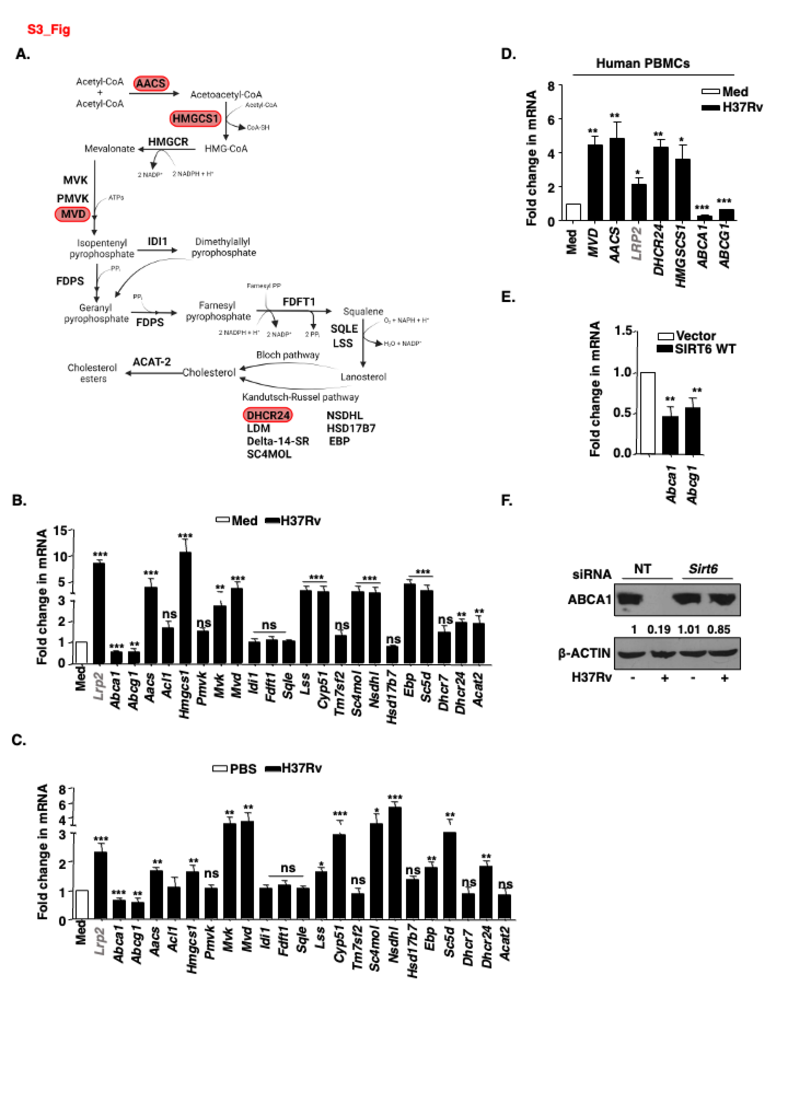

Supplement: S3 Fig — (A) Schematic representation of cholesterol biosynthesis pathway. Transcript level of the indicated set of genes was analyzed by qRT-PCR (B) in BALB/c mouse peritoneal macrophages infected with H37Rv for 12 h, (C) in lung homogenates of BALB/c mice infected with H37Rv for 56 days and (D) in human PBMCs infected with H37Rv for 12 h. (E) RAW 264.7 macrophages were transfected with vector, or SIRT6 WT construct and transcript levels of ABC transporters was analysed by qRT-PCR. (F) BALB/c mouse peritoneal macrophages were transfected with NT or Sirt6, followed by 12 h infection with H37Rv. Whole cell lysates were assessed for ABCA1. The MOI of infection is 1:10 (macrophage:mycobacteria) for all the in vitro experiments. All data represents the mean ± SEM from 3 independent experiments. *, P < 0.05; **, P < 0.01; ***, P < 0.001 (Student’s t-test for B-E). Med, Medium; PBMC, peripheral blood mononuclear cells. (TIF) [file ppat.1011731.s003.tif]

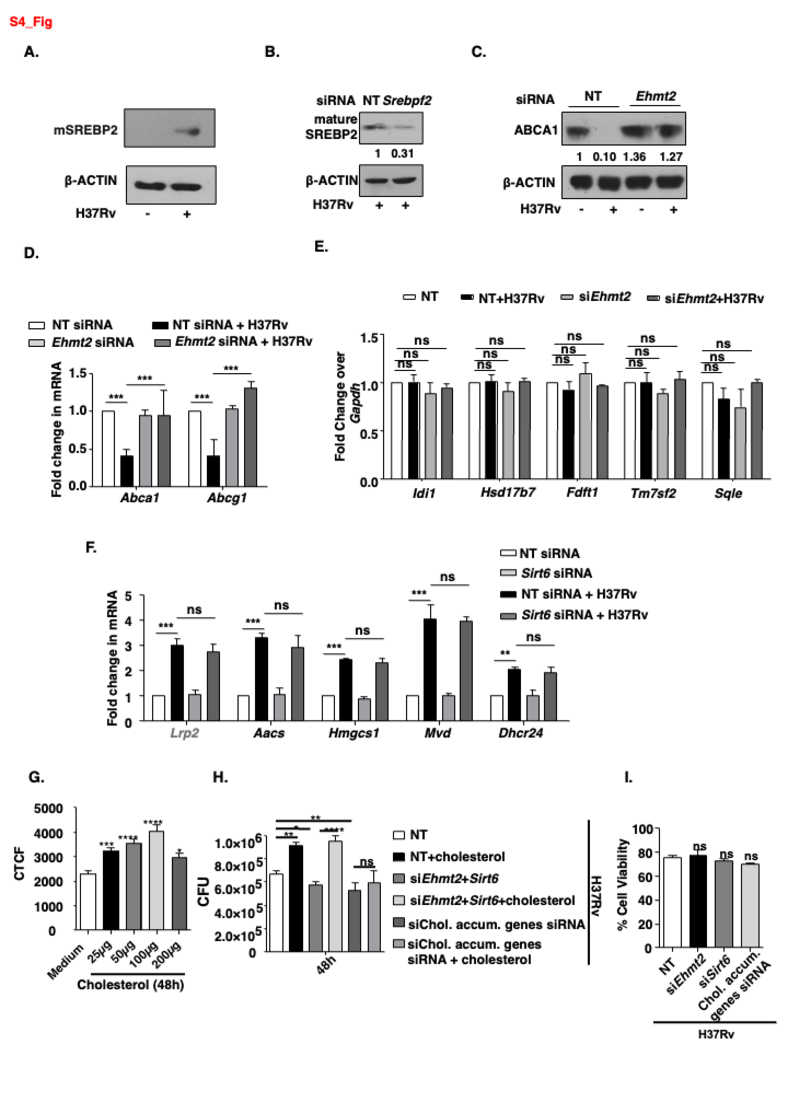

Supplement: S4 Fig — (A) BALB/c mouse peritoneal macrophages were infected with H37Rv for 12 h and whole cell lysates were assessed for mature SREBP2. (B-F) BALB/c mouse peritoneal macrophages were transfected with NT or Ehmt2 or Srebf2 or siSirt6 siRNA as indicated, followed by 12 h infection with H37Rv. Whole cell lysates were assessed for (B) mSREBP2 or (C) ABCA1 expression by immunoblotting. (D-F) transcript level of the indicated genes was measured by qRT-PCR. (G) BALB/c mouse peritoneal macrophages were treated with different concentrations of water-soluble cholesterol for 48h and cholesterol levels were assessed by Filipin staining. (H) In vitro CFU was assessed 48h post H37Rv infection under the following condition: BALB/c mouse peritoneal macrophages transiently transfected with siRNAs against Ehmt2 and Sirt6 or cholesterol accumulation genes (combination of Lrp2, Aacs, Hmgcs1, Mvd and Dhcr24) with and without exogenous cholesterol supplementation(50μg) (I) MTT assay was performed to assess cell viability of BALB/c macrophages transfected with NT or Ehmt2 and Sirt6 or siRNAs against the selected cholesterol accumulation genes (combination of Lrp2, Aacs, Hmgcs1, Mvd and Dhcr24 followed by infection with H37Rv for 48h. The MOI of infection is 1:10 (macrophage: mycobacteria) for all the in vitro experiments. All data represents the mean ± SEM from 3 independent experiments. The blots are representative of 3 independent experiments. *, P < 0.05; **, P < 0.01; ***, P < 0.001 ****, P < 0.0001, ns, not significant (One-way ANOVA for D-H) and ns, not significant (Student’s t-test for I) NT, non-targeting; ns, not significant; mSREBP2, mature SREBP. (TIF) [file ppat.1011731.s004.tif]

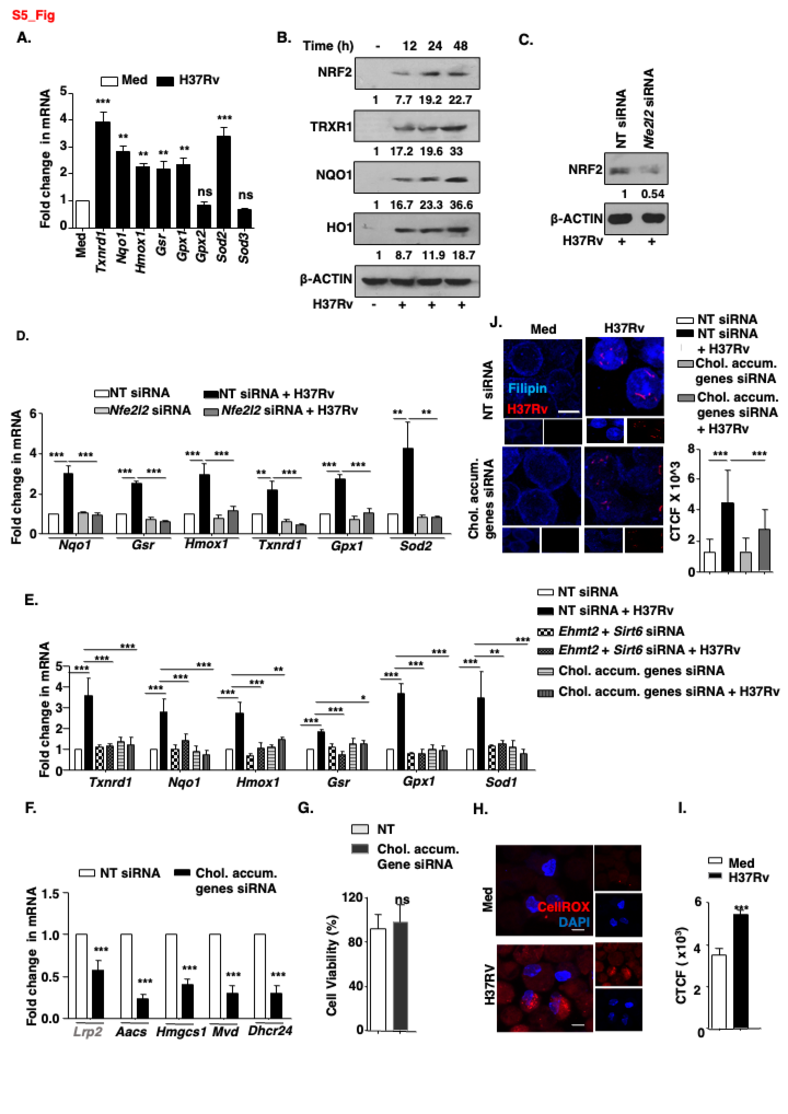

Supplement: S5 Fig — (A) BALB/c mouse peritoneal macrophages were infected with H37Rv for 48 h and the expression of NRF2 target genes was assessed by qRT-PCR. (B) BALB/c mouse peritoneal macrophages were infected with H37Rv for the indicated time points and whole cell lysates were assessed for the expression of NRF2 and its target genes. (C) Immunoblotting to validate NRF2 knockdown in murine macrophages transfected with Nfe2l2 siRNA. (D-F) BALB/c mouse peritoneal macrophages were transfected with NT or (D) Nfe2l2 siRNA or (E) Ehmt2 and Sirt6 siRNA or Chol accum genes siRNA (combination of Lrp2, Aacs, Hmgcs1, Mvd and Dhcr24 siRNAs) or (F) siRNAs against the selected cholesterol accumulation genes (combination of Lrp2, Aacs, Hmgcs1, Mvd and Dhcr24 siRNAs) or NT and assessed for the indicated transcript by qRT-PCR. (G) MTT assay was performed to assess cell viability of BALB/c macrophages transfected with NT or siRNA against selected cholesterol accumulation genes. (H-I) BALB/c mouse peritoneal macrophages were infected with H37Rv for 48 h and stained with CellROX to assess for oxidative stress was performed. Representative images and (H) its quantification (I). (J) BALB/c mouse peritoneal macrophages were transfected with siRNAs against the selected cholesterol accumulation genes (combination of Lrp2, Aacs, Hmgcs1, Mvd and Dhcr24 siRNAs) or NT and followed by H37Rv infection for 48 h and cholesterol accumulation was confirmed by Filipin staining;. The MOI of infection is 1:10 (macrophage:mycobacteria) for all the in vitro experiments. All data represents the mean ± SEM from 3 independent experiments; *, P < 0.05; **, P < 0.01; ***, P < 0.001 (Student’s t- test for A, G, I and One-Way ANOVA for D-F, J) and the blots are representative of 3 independent experiments. Med, Medium; NT, non-targeting; ns, not significant; chol. accum. genes, cholesterol accumulation genes. (TIF) [file ppat.1011731.s005.tif]

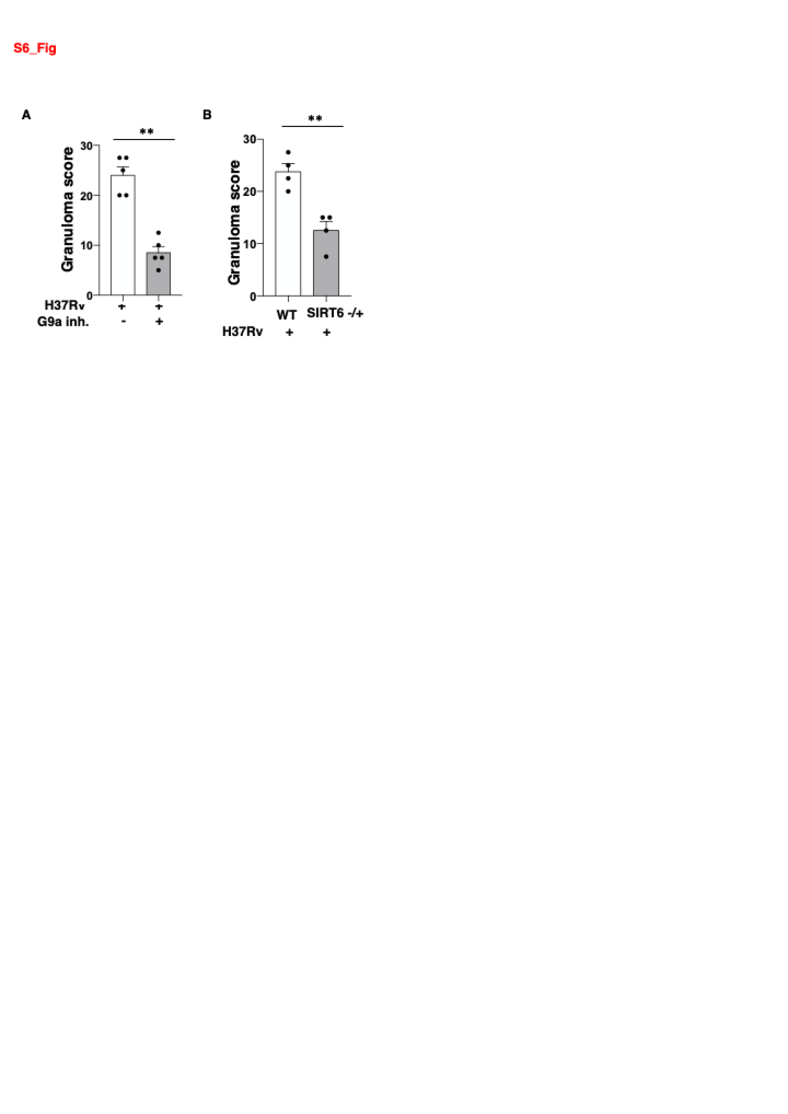

Supplement: S6 Fig — Lungs of BALB/c mice were subjected to histological evaluation for TB pathology by scoring for % of granulomatous area for (A) G9a inhibitor (40mg/kg) treated and untreated BALB/c mice after 56 days of total H37Rv infection and therapeutic treatment and (B) WT (littermate control) and Sirt6−/+ mice. **, p<0.01, Student’s t-test. (TIF) [file ppat.1011731.s006.tif]
