# Supplementary material for: G9a and Sirtuin6 epigenetically modulate host cholesterol accumulation to facilitate mycobacterial survival
Source: PLoS Pathog. 2023 Oct 23;19(10):e1011731. doi: 10.1371/journal.ppat.1011731 (PMC10621959; doi:10.1371/journal.ppat.1011731)
Supplement: S1 File — (PDF) [file ppat.1011731.s007.pdf]

Supplementary File 1

Supplementary File 1a: List of primers for mouse gene expression analyses

Sl. No.	Gene Name	Forward/ Reverse	Sequence (5’-3’)
1.	Glyceraldehyde-3-phosphate dehydrogenase (*Gapdh*)	Forward	gagccaaacgggtcatcatct
		Reverse	gaggggccatccacagtctt
2.	Euchromatic histone lysine N-methyltransferase 2 (*Ehmt2/*G9a)	Forward	agccaagaggggtctccaat
		Reverse	ctcgctgatgcggtcaatct
3.	Sirtuin 6 (*Sirt6*)	Forward	atgtcggtgaattatgcagca
		Reverse	gctggaggactgccacatta
4.	Low density lipoprotein receptor-related protein 2 (*Lrp2*)	Forward	aaaatggaaacggggtgactt
		Reverse	ggctgcatacattgggttttca
5.	ATP-binding cassette, sub-family A (ABC1), member 1 (*Abca1*)	Forward	aaaaccgcagacatccttcag
		Reverse	cataccgaaactcgttcaccc
6.	ATP binding cassette subfamily G member 1 (*Abcg1*)	Forward	gtggatgaggttgagacagacc
		Reverse	cctcgggtacagagtaggaaag
7.	Acetoacetyl-CoA synthetase (*Aacs*)	Forward	gtggaatcgtctactcacgca
		Reverse	taaagggcgactctgtcgttc
8.	ATP citrate lyase (*Acl1*)	Forward	tggatgccacagctgactac
		Reverse	ggttcagcaaggtcagcttc
9.	3-hydroxy-3-methylglutaryl-Coenzyme A synthase 1 (*Hmgcs1*)	Forward	aactggtgcagaaatctctagc
		Reverse	ggttgaatagctcagaactagcc
10.	Phosphomevalonate kinase (*Pmvk*)	Forward	cctatggggctgtgatacaga
		Reverse	tctccgtggttctcaatgacc
11.	Mevalonate kinase (*Mvk*)	Forward	ggtgtggtcggaacttccc
		Reverse	ccttgagcgggttggagac
12.	Mevalonate (diphospho) decarboxylase (*Mvd*)	Forward	ctcagcctcagctataaggtgc
		Reverse	gagccacttcggagaggtc
13.	Isopentenyl-diphosphate delta isomerase (*Idi1*)	Forward	agcttctagcggagatgtgta
		Reverse	cagcaactattggtgaaacaacc
14.	Farnesyl diphosphate farnesyl transferase 1 (*Fdft1*)	Forward	gtttgaagaccccatagttggtg
		Reverse	cacatctacgttctctggcttag
15.	Squalene epoxidase (*Sqle*)	Forward	ataagaaatgcggggatgtcac
		Reverse	atatccgagaaggcagcgaac
16.	Lanosterol synthase (*Lss*)	Forward	gggaaggactcaacaccctat
		Reverse	cgtagcagtaactcatgggca
17.	Cytochrome P450, family 51 (*Cyp51*)	Forward	aacgaagacctgaatgcagaag
		Reverse	gtgggctatgttaaggccact
18.	Transmembrane 7 superfamily member 2(*Tm7sf2*)	Forward	ggcctttgcgaccactctc
		Reverse	gttcagctcccgtccaagaaa
19.		Forward	tcatcggaattgtgcttttgtgt

	Methylsterol monooxygenase 1 (*Sc4mol*)	Reverse	cagcgggttgagaggaatatc
20.	NAD(P) dependent steroid dehydrogenase-like (*Nsdhl*)	Forward	acgccatgaagcctattgact
		Reverse	ggtcccttgggccgaaaat
21.	Hydroxysteroid (17-beta) dehydrogenase 7 (*Hsd17b7*)	Forward	tctctgccatgtggataaccc
		Reverse	ggtcggtagcgtatttggaag
22.	Phenylalkylamine Ca2^{+} antagonist (emopamil) binding protein (*Ebp*)	Forward	actggccttgtgctggttt
		Reverse	tccatacagacgacgaagctg
23.	Sterol-C5-desaturase (*Sc5d*)	Forward	ggggttacagcaaactctacg
		Reverse	ggtgcaggcccctatgaat
24.	7-dehydrocholesterol reductase (*Dhcr7*)	Forward	cagatttctgccaggttatgtgg
		Reverse	agaaccaggataagaggtaagcg
25.	24-dehydrocholesterol reductase (*Dhcr24*)	Forward	gcacaggcatcgagtcatc
		Reverse	cagggcacggcatagaaca
26.	Acetyl-Coenzyme A acetyltransferase 2 (*Acat2*)	Forward	tccattcaaaacatggggat
		Reverse	tcagcctggaagaggtcact
27.	Thioredoxin reductase 1 (*Txnrd1*/TRXR1)	Forward	cccacttgccccaactgtt
		Reverse	gggagtgtcttggagggac
28.	NAD(P)H dehydrogenase, quinone 1 (*Nqo1*)	Forward	ttctctggccgattcagagt
		Reverse	ggctgcttggagcaaaatg
29.	Heme oxygenase 1 (*Hmox1*/HO-1)	Forward	cacgcatatacccgctacct
		Reverse	ccagagtgttcattcgaga
30.	Glutathione reductase (*Gsr*)	Forward	gacacctcttccttcgactacc
		Reverse	cccagcttgtgactctccac
31.	Glutathione peroxidase 1 (*Gpx1*)	Forward	gtccaccgtgtatgccttct
		Reverse	tctgcagatcgttcatctcg
32.	Glutathione peroxidase 2 (*Gpx2*)	Forward	gcctcaagtatgtccgacctg
		Reverse	ggagaacgggtcatcataaggg
33.	Superoxide dismutase 2 (*Sod2*)	Forward	gcggtcgtgtaaacctcat
		Reverse	ccagagcctcgtggtacttc
34.	Superoxide dismutase 3 (*Sod3*)	Forward	ctgaggacttcccagtgac
		Reverse	ggtgagggtgtcagagtgt

Supplementary File 1b: List of primers for human gene expression analyses

Sl. No.	Gene Name	Forward/ Reverse	Sequence (5’-3’)
1.	Glyceraldehyde-3-phosphate dehydrogenase(*GAPDH*)	Forward	ggagcgagatccctccaaaat
		Reverse	ggctgttgtcatacttctcatgg
2.	Euchromatic histone lysine N-methyltransferase 2 (*EHMT2*/G9a)	Forward	gggcgggaaaatcacctcc
		Reverse	cactcatgcggaaatgctgtat
3.	Sirtuin 6 (*SIRT6*)	Forward	cccacggagtctggaccat
		Reverse	ctctgccagtttgtccctg
4.		Forward	gttcagatgacgcggatgaaa

	Low density lipoprotein receptor-related protein 2 (*LRP2*)	Reverse	tcacagtcttgatcttggtcaca
5.	ATP-binding cassette, sub-family A (ABC1), member 1 (*ABCA1*)	Forward	ttcccgcattatctggaaagc
		Reverse	caaggtccatttcttggctgt
6.	ATP binding cassette subfamily G member 1 (*ABCG1*)	Forward	cgtgcgctttgtgctgttt
		Reverse	ccactgtaggtacgtggggat
7.	Acetoacetyl-CoA synthetase (*AACS*)	Forward	ggcagtcggctcaactatg
		Reverse	acaacccgatctcctttcttca
8.	3-hydroxy-3-methylglutaryl-Coenzyme A synthase 1 (*HMGCS1*)	Forward	gatgtgggaattgttgccctt
		Reverse	attgtctctgttccaacttccag
9.	Mevalonate (diphospho) decarboxylase (*MVD*)	Forward	ggaccggatttggctgaatg
		Reverse	cccatcccgtgagttcctc
10.	24-dehydrocholesterol reductase (*DHCR24*)	Forward	cactgtctcactacgtgtcgg
		Reverse	ccagccaatggaggtcagc

Supplementary File 1c: List of primers for ChIP assays

Sl. No.	Gene Name		Sequence
	**For G9a binding and Sequential ChIP**		
1.	*Lrp2*	Forward	aggcacaggtcgaggatct
		Reverse	ctgccctccagtctcagttt
2.	*Aacs*	Forward	tgctaccgtttcgttcactg
		Reverse	gcaggtttccgaacaaagag
3.	*Hmgcs1*	Forward	cattggcaggcttgttctc
		Reverse	gatccgctttcagccaatg
4.	*Mvd*	Forward	aaaagcaactcccattcactg
		Reverse	tggctgttgaatggcttagag
5.	*Dhcr24*	Forward	ctcccactctagggaatcca
		Reverse	cagtgctattgcaggtgttca
	**For SIRT6 binding and Time Kinetics ChIP**		
6.	*Abca1*	Forward	agtccggagtttcccgttt
		Reverse	agcagaaagcacgtggagac
7.	*Abcg1*	Forward	ccgactaggccatcttttga
		Reverse	agctaatggatggatcacagg
	**For β-CATENIN binding**		
8.	*Ehmt2*	Forward	atgtcctcatccgctgaaag
		Reverse	gtctccggctccatcttttt
		Forward	agttcagcagctcacacagg

9.	*Sirt6*	Reverse	gaacttggaagctccgtttg
