# Supplementary material for: G9a and Sirtuin6 epigenetically modulate host cholesterol accumulation to facilitate mycobacterial survival
Source: PLoS Pathog. 2023 Oct 23;19(10):e1011731. doi: 10.1371/journal.ppat.1011731 (PMC10621959; doi:10.1371/journal.ppat.1011731)
Supplement: S2 File — (PDF) [file ppat.1011731.s008.pdf]

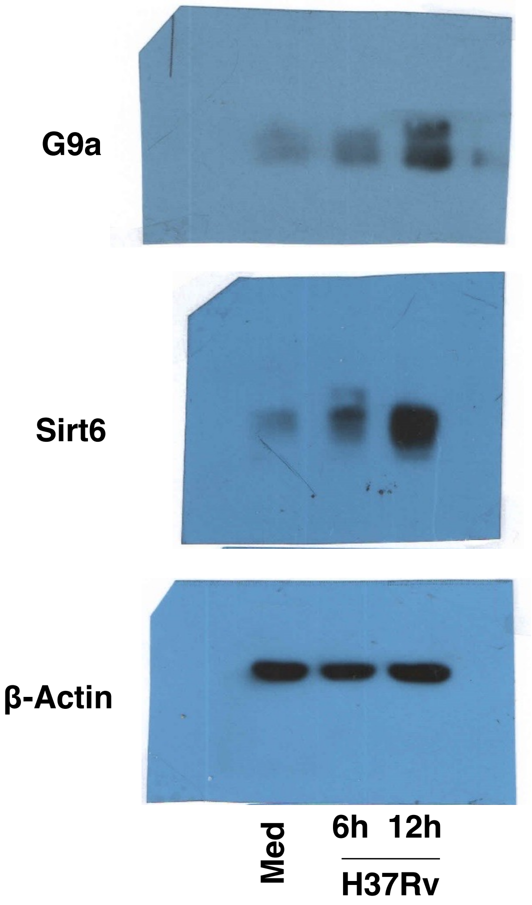

Figure1_A

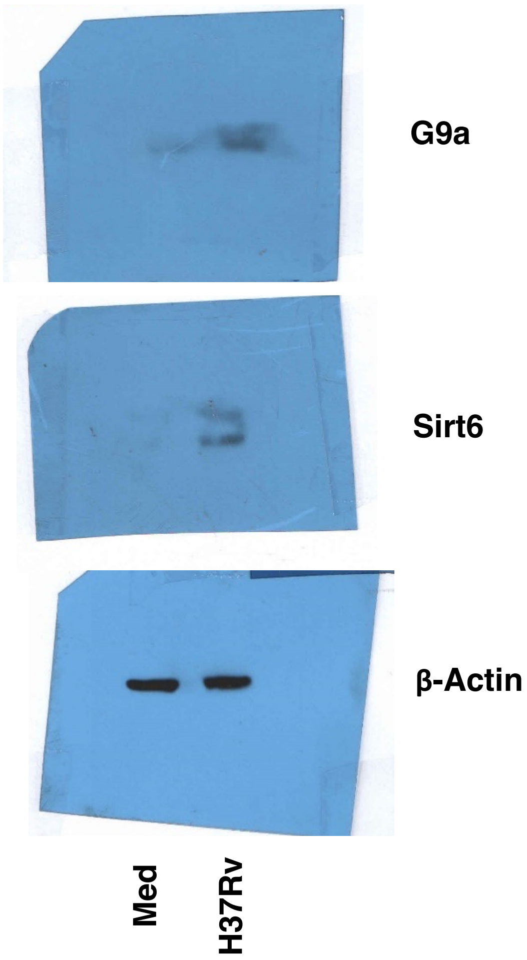

Figure1_E

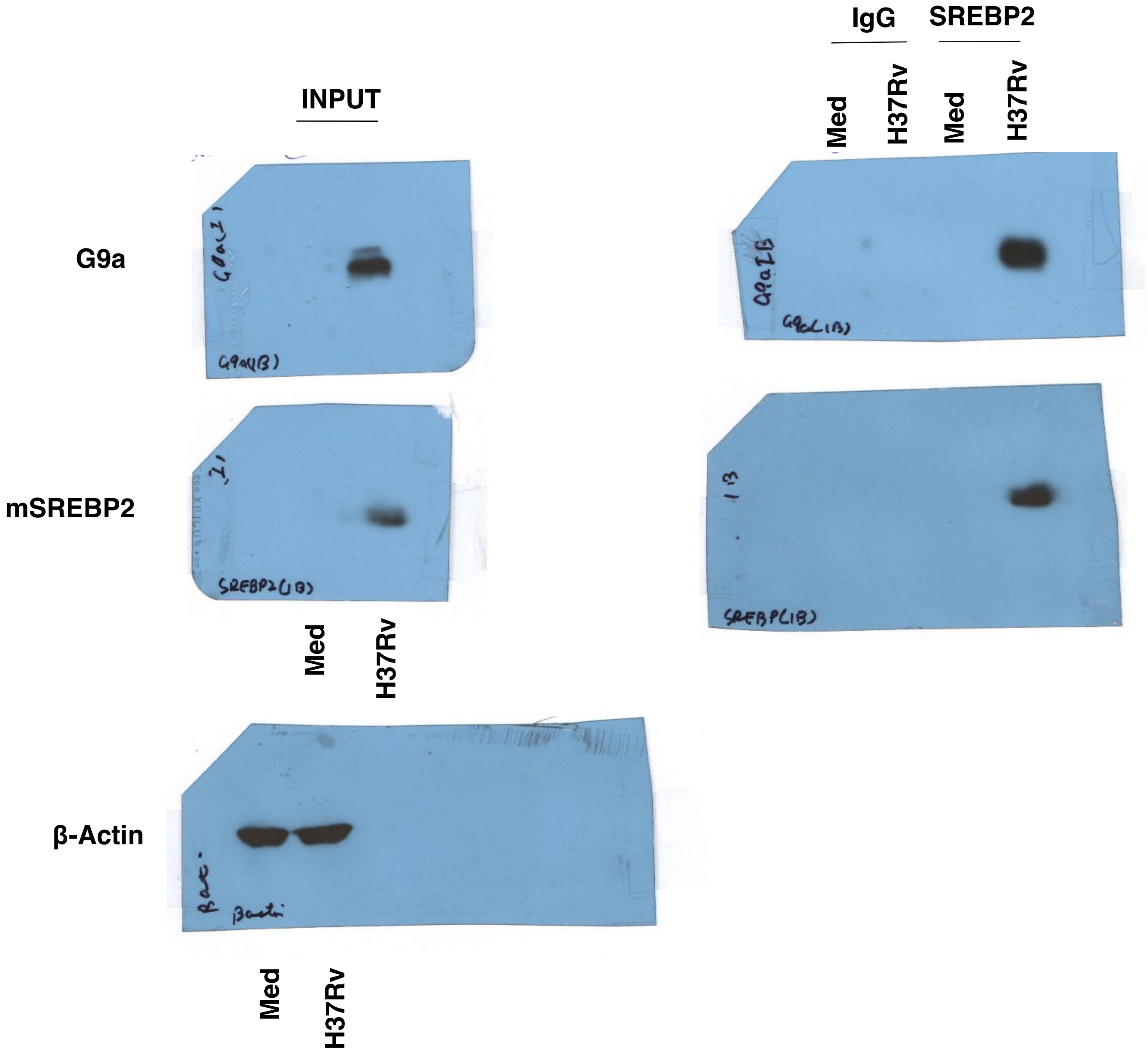
INPUT
IgG
SREBP2
Med
H37Rv
Med
H37Rv
G9a
mSREBP2
Med
H37Rv
β-Actin
Med
H37Rv

Figure3_A

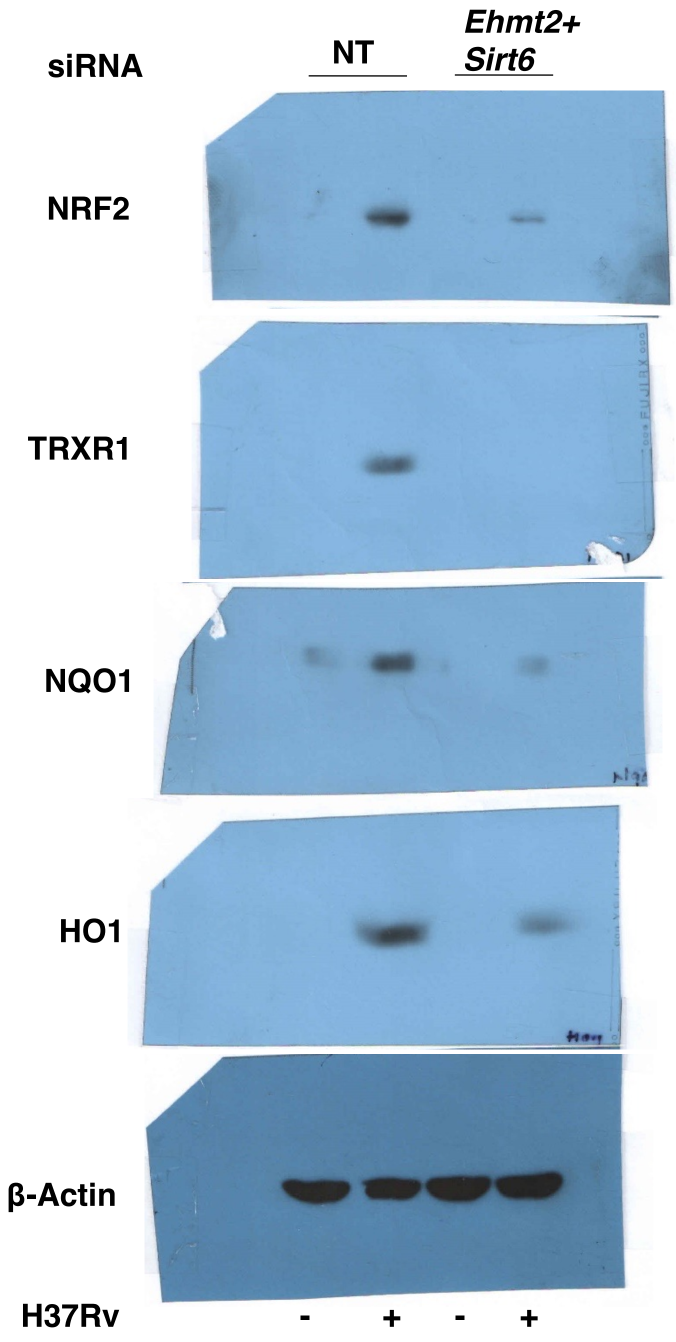

Figure4_A

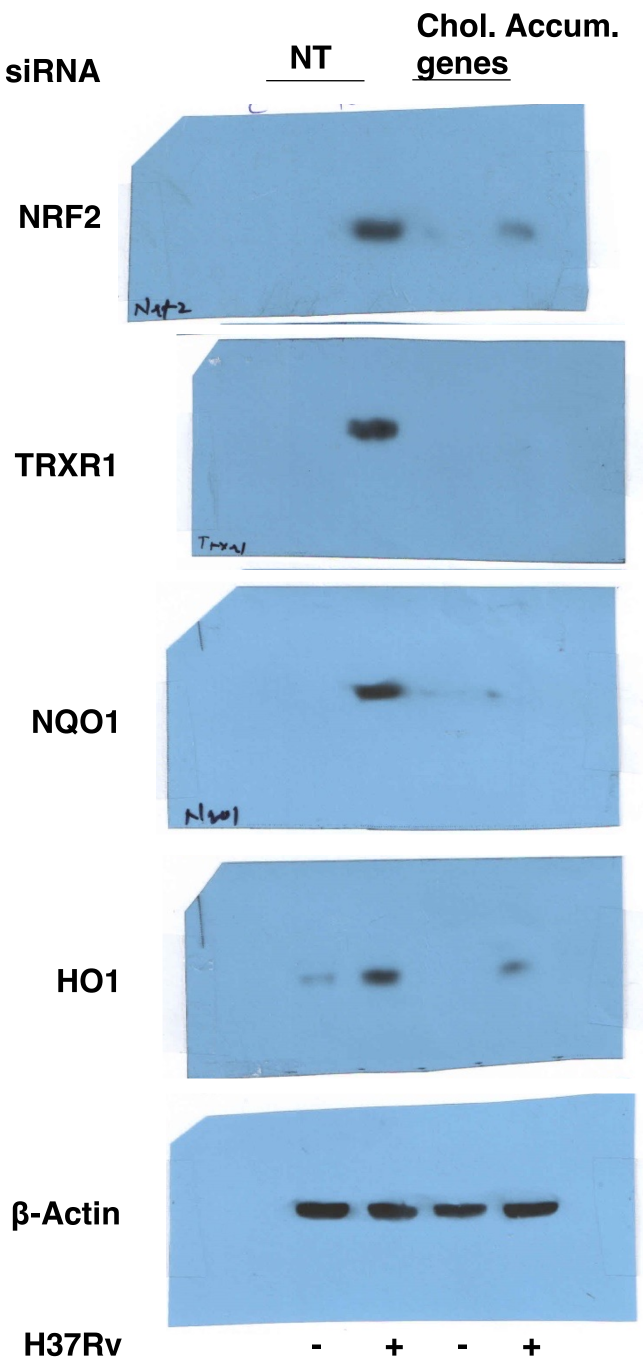

Figure4_B

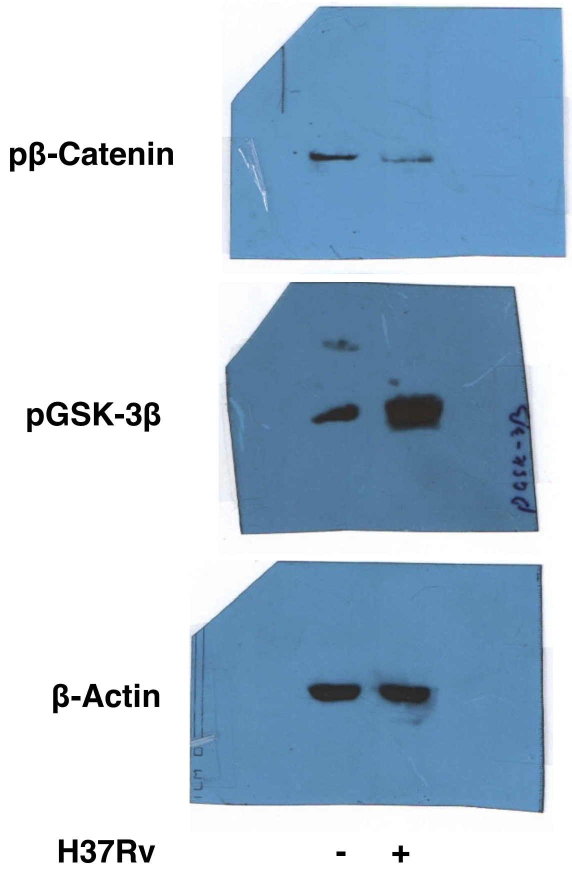

Figure5_A

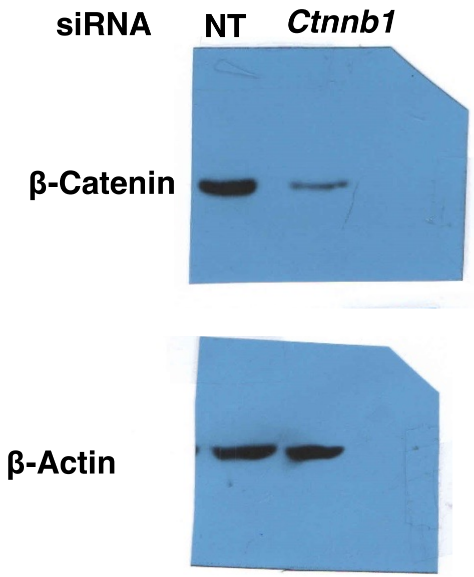

Figure5_B

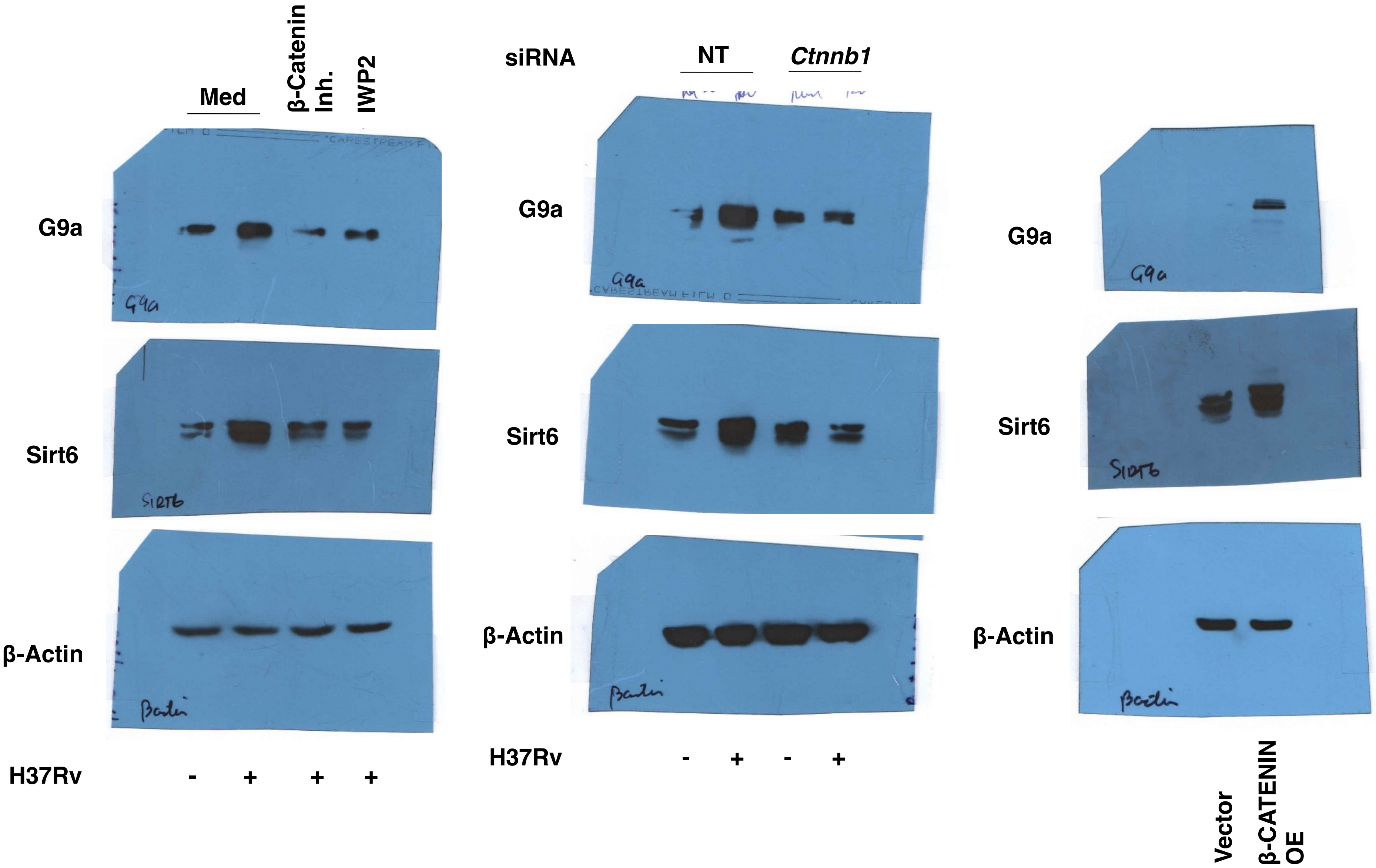
Med
β-Catenin Inh.
IWP2
siRNA
NT
Ctnnb1
G9a
Sirt6
β-Actin
H37Rv
- + + +
G9a
Sirt6
β-Actin
H37Rv
- + - +
G9a
Sirt6
β-Actin
Vector
β-CATENIN OE

Figure5_C

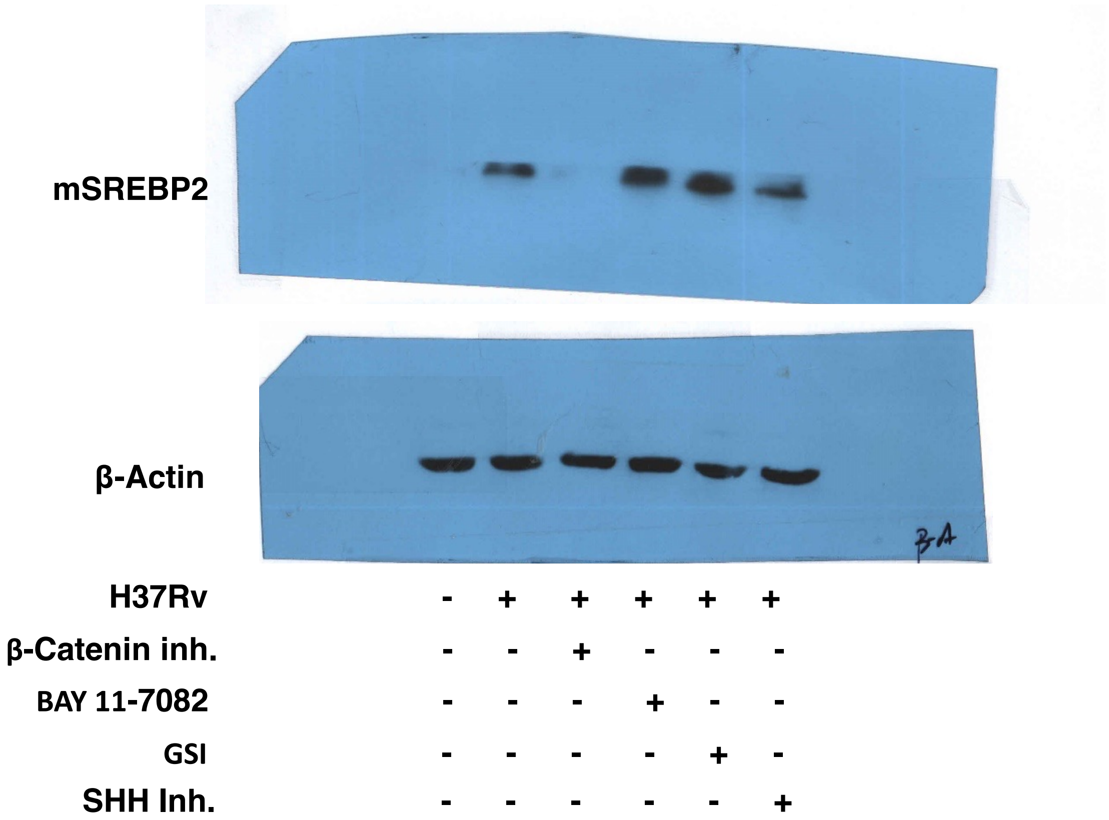

Figure5_E

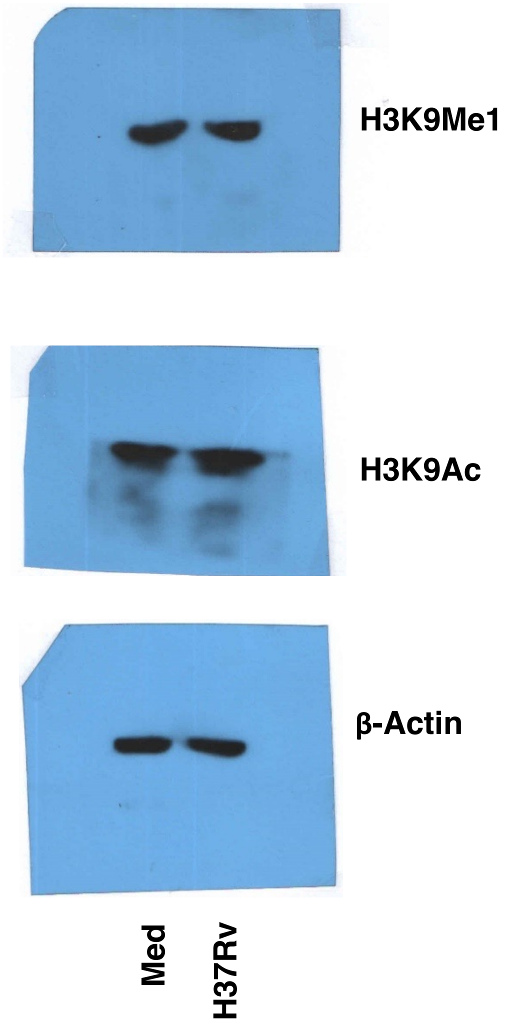

Supplementary Figure 1_A

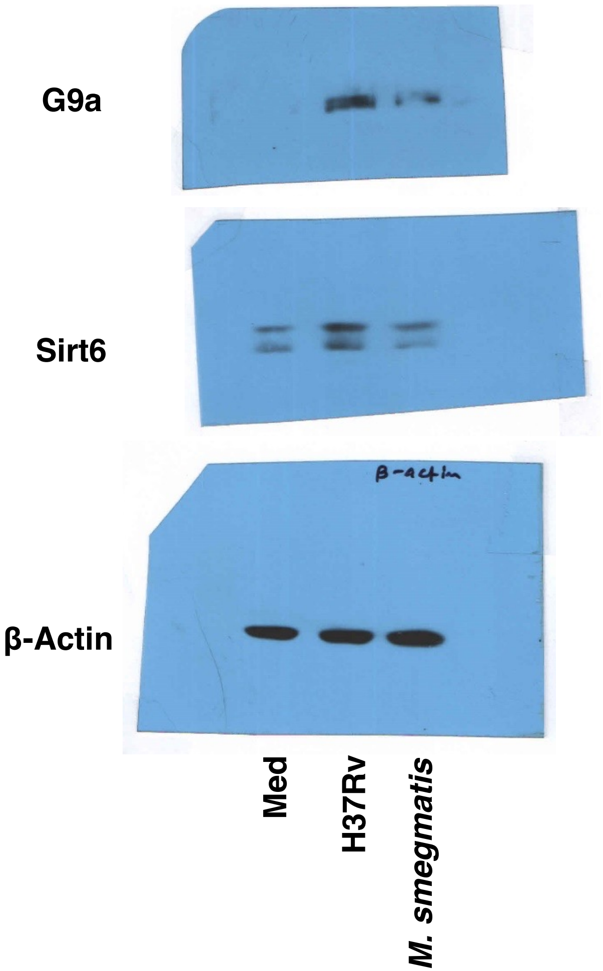

Supplementary Figure 1_C

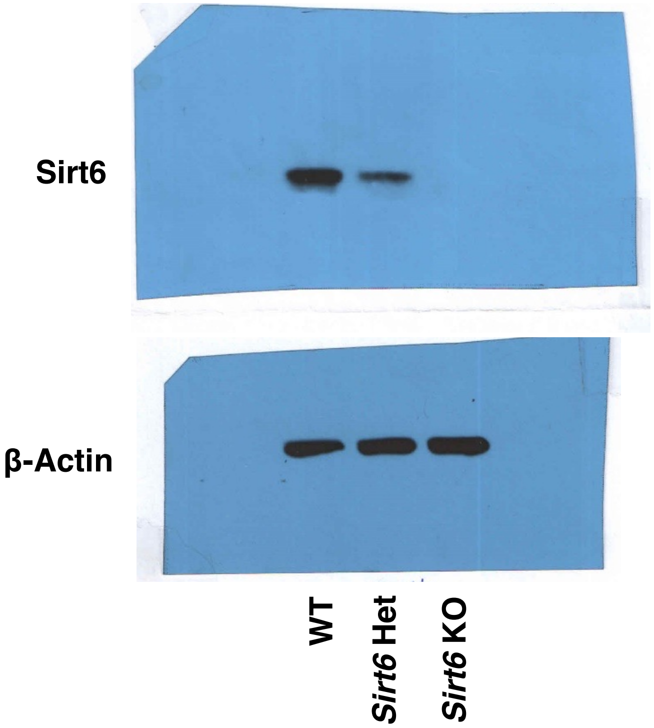

Supplementary Figure 1_D

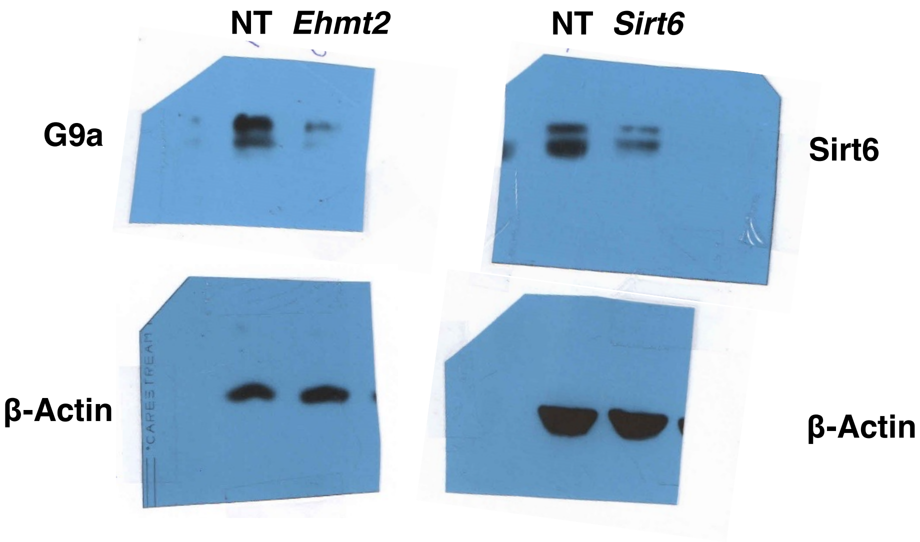

Supplementary Figure 1_E

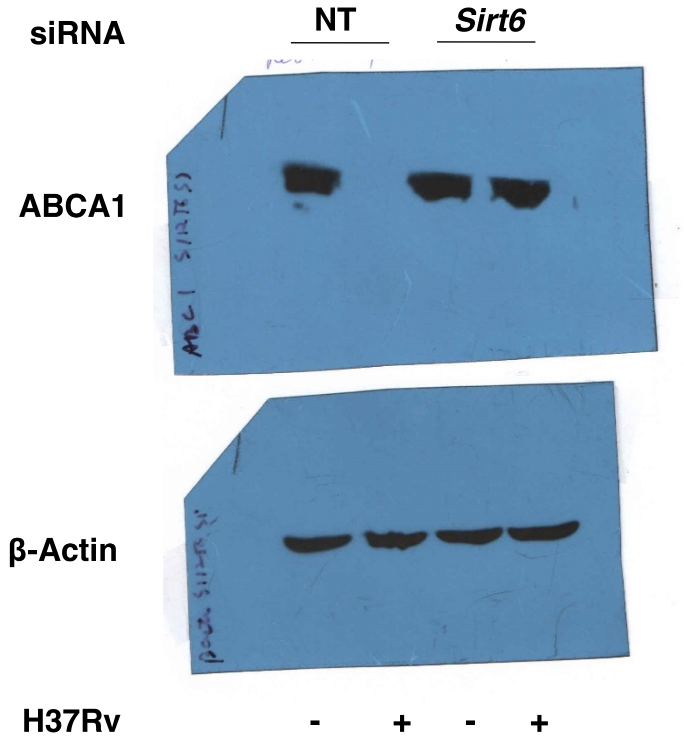

Supplementary Figure 3_F

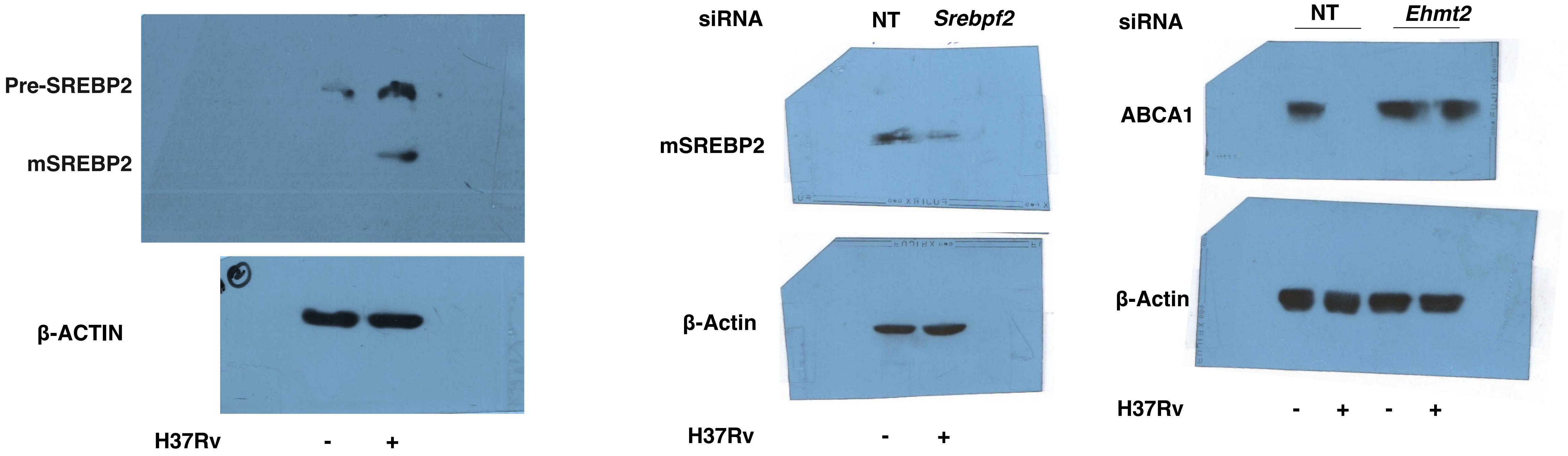

Supplementary Figure 4_A-C

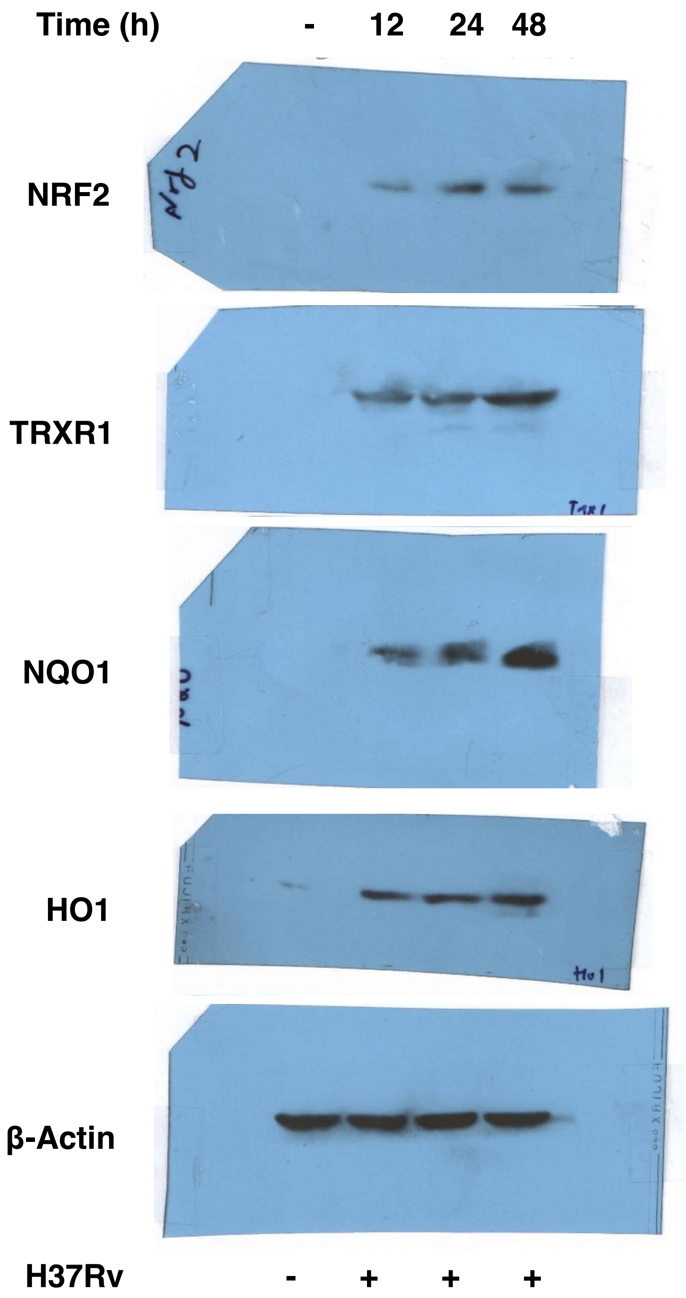

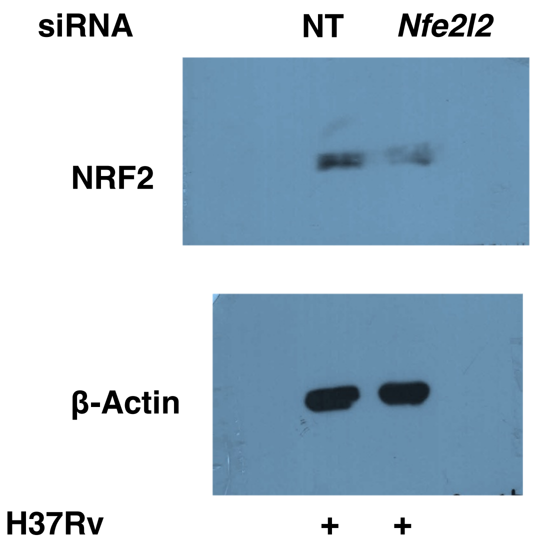

Supplementary Figure 5_B-C
